# Mixed-Effects Location Scale Models for Joint Modeling School Value-Added Effects on the Mean and Variance of Student Achievement

**DOI:** 10.3102/10769986231210808

**Published:** 2023-11-27

**Authors:** George Leckie, Richard Parker, Harvey Goldstein, Kate Tilling

**Affiliations:** University of Bristol

**Keywords:** school value-added models, mixed-effect models, mixed-effects location scale models, school effectiveness, school accountability

## Abstract

School value-added models are widely applied to study, monitor, and hold schools to account for school differences in student learning. The traditional model is a mixed-effects linear regression of student current achievement on student prior achievement, background characteristics, and a school random intercept effect. The latter is referred to as the school value-added score and measures the mean student covariate-adjusted achievement in each school. In this article, we argue that further insights may be gained by additionally studying the variance in this quantity in each school. These include the ability to identify both individual schools and school types that exhibit unusually high or low variability in student achievement, even after accounting for differences in student intakes. We explore and illustrate how this can be done via fitting mixed-effects location scale versions of the traditional school value-added model. We discuss the implications of our work for research and school accountability systems.

## 1 Introduction

School value-added models attempt to estimate school differences in student achievement and are widely applied in educational (Goldstein, 1997; Reynolds et al., 2014; Teddlie & Reynolds, 2000) and statistical research (American Statistical Association, 2014; Braun & Wainer, 2007; McCaffrey et al., 2004; Raudenbush & Willms, 1995; Wainer, 2004). They are also used in the United States, United Kingdom, and other school accountability systems, where the predicted school differences, often referred to as school value-added scores, provide the basis of reward and sanction decisions on schools (Amrein-Beardsley, 2014; Castellano & Ho, 2013; Koretz, 2017; Leckie & Goldstein, 2017; Organization for Economic Cooperation and Development, 2008). In educational and statistical research, there is an additional interest in identifying school policies and practices that predict the school differences and that might therefore prove effective at raising student achievement in schools in general.

The traditional school value-added model is formulated as a mixed-effects (multilevel or hierarchical) linear regression model (Goldstein, 2011; Raudenbush & Bryk, 2002; Snijders & Bosker, 2012) of student current achievement on student prior achievement measured at the start of the value-added period (typically defined as one or more school years or a phase of schooling) and a school random intercept effect to predict the school differences (Aitkin & Longford, 1986; Goldstein et al., 1993; Raudenbush & Bryk, 1986). The adjustment for student prior achievement is fundamental as simpler comparisons of unadjusted school mean achievement would in large part reflect school differences in student achievement present at the start of the value-added period. Such differences are argued beyond the control of the school. Student sociodemographic characteristics are often added to adjust for initial school differences in student composition more convincingly (Ballou et al., 2004; Leckie & Goldstein, 2019; Leckie & Prior, 2022; Levy et al., 2023). Schools with higher scores are described as adding more value: producing higher student achievement for any given set of students. The scores are argued to reflect the net influences of differences in the quality of teaching, availability of resources, and other policies and practices across schools, which are typically unobserved to the data analyst. The regression coefficient on student prior achievement is occasionally allowed to vary across schools. The resulting random slope model is sometimes referred to as a “differential school effectiveness” model as this extension allows schools to now have different effects for different types of students (Nuttal et al., 1989; Scherer & Nilsen, 2019; Strand, 2010).

While the traditional school value-added model is widely applied (Levy et al., 2019), it is important to realize that this model is just a regression model fitted to observational data and so the effects attributed to schools may also be caused by other factors that are not captured by the model (American Statistical Association, 2014). That is, while there is consensus that the predicted school effects are fairer and more meaningful measures to compare schools than comparing simple school mean achievement scores, the additional assumptions required to interpret these predicted school effects as causal effects rather than as merely adjusted school mean differences are challenging (Amrein-Beardsley, 2019; Reardon & Raudenbush, 2009; Rubin et al., 2004). For example, the school-level exogeneity assumption (independence of covariates and school random effect) will fail if higher prior achieving students select into more effective schools, perhaps because such students are from more affluent families who are more able to buy into the catchment areas of these schools (Angrist et al., 2021; De Fraine, 2002; Thomas & Mortimore, 1996; Timmermans & Thomas, 2015). The parameter estimates of the school value-added models presented in this article should therefore be viewed as the measures of association and the predicted school effects as descriptive differences in means and variances of student achievement across schools, where inevitably only partial and imperfect adjustments have been made for school differences in student characteristics at intake.

In the traditional school value-added model, the difference between observed and predicted student current achievement defines the total residual, which can be viewed as a covariate-adjusted (residualized) measure of student current achievement (i.e., a controlled comparison of student achievement levels). The total residual is modeled as the summation of the school random intercept effect and the student residual. The school random effect measures the mean student adjusted achievement in each school. In contrast, the constant residual variance implicitly assumes the variance in student adjusted achievement is the same in every school. This inconsistent modeling of the mean and variance does not seem realistic. Any given school policy or practice will have different effects on students as a function of their observed and unobserved characteristics and will therefore contribute to the variance in student adjusted achievement operating in each school. Indeed, this is the motivation for the random slope extension to the traditional value-added model described above. In practice, however, this extension can only be used to account for a limited number of observed student characteristics, not to all observed and unobserved student characteristics (Raudenbush & Bryk, 2002). Thus, the different sets of school policies and practices operating in each school will lead the variance in student adjusted achievement to vary across schools, even in random slope models.

Studying the variance in student adjusted achievement in each school may therefore provide valuable new insights into the differences in student learning between schools. Consider two schools that show similarly high levels of mean student adjusted achievement. The traditional school value-added model would describe these two schools as equally effective. Suppose, however, the first school shows higher variance in their student adjusted achievement scores than the second school. Which school should now be viewed more positively? The school with the higher variance will have more students making exceptionally high adjusted achievement (a positive) albeit at the expense of more students also making unacceptably low adjusted achievement (a negative). All else equal, the school with the higher variance will also show a weaker link between prior and current achievement, and so in this school, low prior achievement students are more able to raise up the achievement distribution (a positive), but equally and necessarily, high prior achievement students are more likely to fall down the distribution (a negative). Thus, in part, how higher variance should be viewed depends on value judgements regarding whether such positives outweigh such negatives. These are not simple questions to answer. Also relevant is the underlying explanation for the difference in variance. For example, if the higher variance seen in the first school is a result of its school policies and practices having greater differential effects on different student groups versus the second school, then higher variance might be viewed as a negative as the explanation implies that the school might not be in sufficient control in the implementation of its policies and practices and is exacerbating inequities in student learning versus the first school (Nuttal et al., 1989; Scherer & Nilsen, 2019; Strand, 2010). Though, here too, a tension lies around what is the optimal level of control. Again, these are not simple questions to answer. More generally, school differences in the adjusted variances, just like school differences in the adjusted means, may also reflect unmodeled school differences in student intake, and so, it is important to attempt to adjust fully for such differences.

A necessary first step to addressing these bigger questions and debates is to first measure school differences in the variance in student adjusted achievement. Only then can school effectiveness and other researchers follow up individual schools, which show unusually high or low variance to try to identify the specific school policies and practices, which are associated with this. Similarly, only then, can school accountability systems, via school inspections, ask schools to reflect on any unusual school variance scores and discuss these within the broader context of what is happening in these schools and other schools facing similar challenges. All these discussions should be alert to the descriptive rather than causal nature of the statistics and to the limitations of the data more generally, and these statistics should not be used to make automatic high-stakes judgements on schools.

The aim of this article is to therefore broaden the traditional school value-added model to study the effects of schools on not just mean student current achievement, but the variance in student current achievement. We do this by applying mixed-effect location scale (MELS) models to student current achievement. MELS models are an extension to conventional mixed-effects linear regression models that model the residual variance not as a constant, but as a function of the covariates and a new random effect. Thus, the residual variance is now allowed to vary across the schools. Hedeker et al. (2008) illustrated the MELS model in the context of studying intensive longitudinal data on mood. Subsequently, Hedeker and others further developed this class of models and applied it to a range of other longitudinal psychological and health data (e.g., Goldstein et al., 2018; Hedeker et al., 2012; Nordgren et al., 2020; Parker et al., 2021; Rast et al., 2012). Just as mixed-effects models more generally are routinely also applied to clustered cross-sectional data, so can MELS models. Indeed, several such applications have now been published, including in social science research (Brunton-Smith et al., 2017, 2018; Leckie et al., 2014; McNeish, 2021). However, the applicability of MELS models to school value-added studies has not yet been explored. We address this via an application to school value-added models for school accountability in London, England. Specifically, we examine the following research question: *How does the variance in student adjusted achievement vary across schools?*

This article proceeds as follows. In Section 2, we introduce our application. In Section 3, we present the traditional random-intercept and -slope linear regression school value-added models and their extensions to MELS models. In Section 4, we present the results. In Section 5, we provide a general discussion, including implications of our work for research and school accountability.

## 2 Application

### Background

In England, since 2004, the Government has published school value-added scores derived from school value-added models for all secondary schools in the country in annual school performance tables (https://www.gov.uk/school-performance-tables). These scores aim to measure the value that each school adds to student achievement between the end of primary schooling national Key Stage 2 (KS2) tests (age 11, academic year 6) and the end of compulsory secondary schooling General Certificate of Secondary Education (GCSE) examinations (age 16, academic year 11). The scores play a pivotal role in the national school accountability system, informing school inspections and judgments on schools. They are also promoted to parents as a source of information when choosing schools for their children. Their high stakes use and public presentation have drawn sustained criticism from the academic literature (Goldstein & Spiegelhalter, 1996; Leckie & Goldstein, 2009, 2017, 2019; Prior, Jerrim, et al., 2021). Nevertheless, these authors also argue that when used carefully and collaboratively with schools in a sensitive and less public manner, there is still an important role for these scores to help identify and understand differences in student outcomes across schools, and it is in this spirit that we have carried out the current research (Goldstein, 2020).

### Data, Sample, and Variables

We focus on schools in London and on those students who took their GCSE examinations in 2018 and therefore KS2 tests in 2013. The sample is drawn from the National Pupil Database (Department for Education [DfE], 2023) a census of all students in state education and consists of 71,321 students in 465 schools (mean = 153 students per school, range = 14–330).

**Figure 1:**
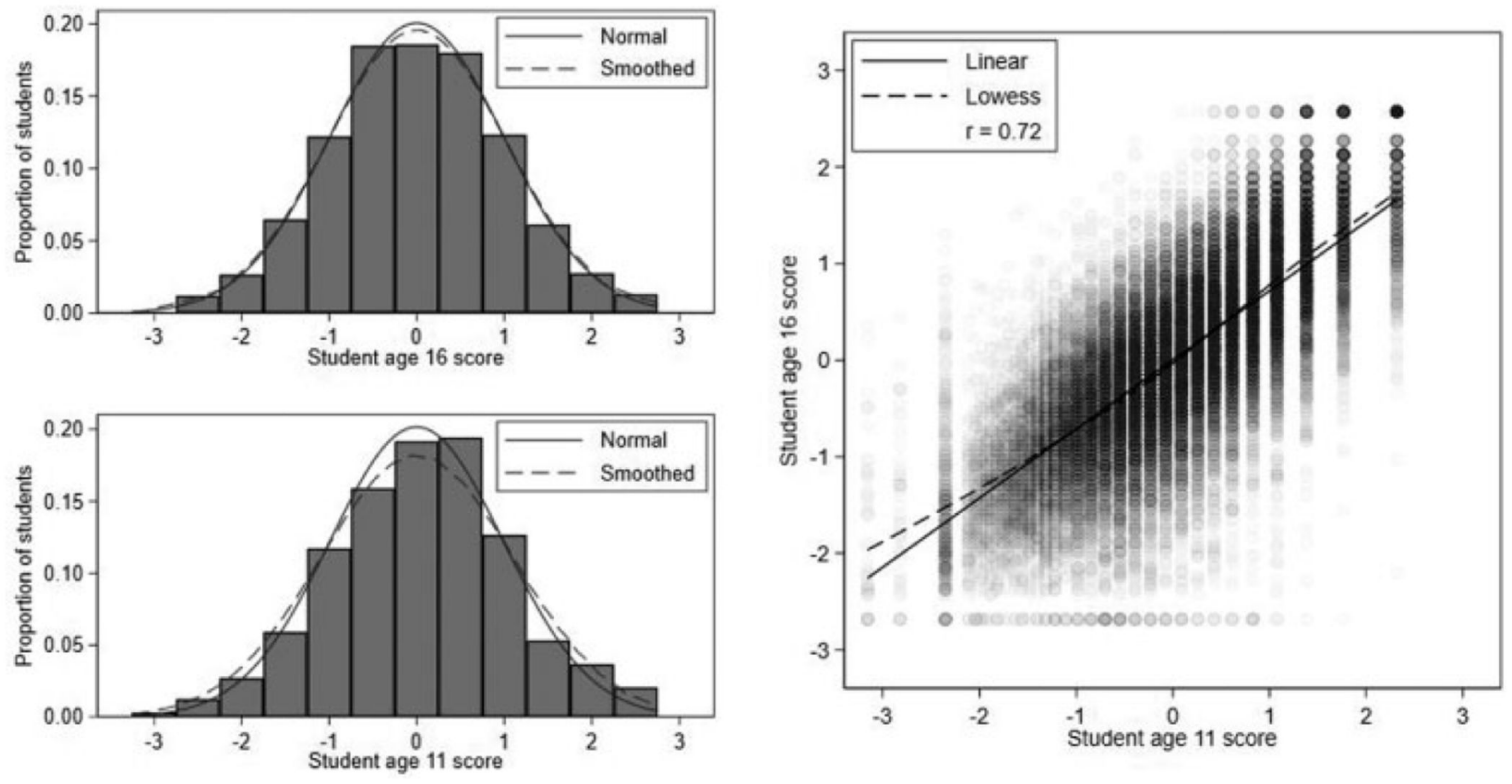
Histograms and scatterplot of student age 16 and age 11 scores.

Student current and prior achievement are measured by students’ GCSE examination and KS2 test scores (DfE, 2020). We standardize these scores to have means of 0 and standard deviations (*SD*s) of 1, so that the measures can be interpreted in *SD* units. Henceforth, we refer to these standardized scores simply as the student age 16 and 11 scores. Figure 1 shows both scores are approximately normally distributed and linearly related with a strong Pearson correlation of 0.72. There are very slight floor and ceiling effects in age 16 scores.

Table 1 presents the summary statistics for the student characteristics. Of note, 61% of students are non-White and 35% poor (as measured by receipt of free school meals [FSMs]). The London sample is therefore more ethnically diverse and poorer than the full English sample, where only around 25% of students are non-White and 25% poor (Leckie & Goldstein, 2019).

Table 2 presents the summary statistics for the school characteristics. A range of school types operate in London (Leckie & Goldstein, 2019), and we have categorized these into four groups: standard, sponsored academy, converter academy, and other. Standard school type encompasses community, foundation, voluntary aided, voluntary controlled, and city technology colleges. In contrast to standard and other schools, academies receive their funding directly from the government rather than through local authorities (school districts). Sponsored academies are mostly underperforming schools, which have been required to change to academy status and are run by sponsors. Converter academies are successfully performing schools that have opted to convert to academy status. Other school type encompasses free, studio, university technology colleges (UTCS), and further education colleges. These are more technically or vocationally oriented schools.

A minority of local authorities operate selective rather than comprehensive admissions. In these areas, grammar schools select students based on high performance in entrance examinations and so by definition have high mean age 11 scores and tend also to be educationally advantaged and homogenous in terms of student sociodemographic characteristics. Secondary modern schools take those students not admitted to grammar schools.

## 3 Models

### Model 1: Random-Intercept Model

The traditional school value-added model (Aitkin & Longford, 1986; Goldstein et al., 1993; Raudenbush & Bryk, 1986) can be written as the following random-intercept linear regression:

(1) $$\begin{array}{l} y_{ij}=\beta_{0}+\beta_{1}x_{1ij}+u_{j}+e_{ij}\\ u_{j}\sim N\left(0,\sigma_{u}^{2}\right),\\ e_{ij}\sim N\left(0,\sigma_{e}^{2}\right), \end{array}$$

where *y*_*ij*_ and *x*_1*ij*_ denote current and prior achievement for student *i* (*i* = 1, …, *n*_*j*_) in school *j* (*j* = 1, …, *J*), β_0_ and β_1_ denote the regression coefficients, *u*_*j*_ the school random intercept effect, *e*_*ij*_ the student residual, and where *u*_*j*_ and *e*_*ij*_ are assumed independent of one another, independent of *x*_1*ij*_, and normally distributed with zero means and constant variances $\sigma_{u}^{2}$ and $\sigma_{e}^{2}$. As discussed in Section 1, the independence assumptions are unlikely to hold, and so in this article, we interpret the school value-added model and the predicted school effects as descriptive rather than causal. Further student and school covariates may be added to this model, and we will explore this in Section 4.

The total residual *u*_*j*_ *+ e*_*ij*_ measures covariate-adjusted (residualized) student current achievement. That is student current achievement having adjusted for prior achievement. The overall average adjusted achievement is 0. The random effect *u*_*j*_ therefore measures the mean student adjusted achievement in each school (the traditional school value-added score), while the residual *e*_*ij*_ measures the adjusted achievement of each student relative to their school mean. The random effect variance $\sigma_{u}^{2}$ measures the variation in school mean adjusted achievement across schools. The residual variance $\sigma_{e}^{2}$ measures the average variance in student adjusted achievement within schools. Crucially, this parameter is averaged across all schools. Thus, while the model allows mean student adjusted achievement to vary from school to school *u*_*j*_, it assumes the variance in student adjusted achievement is the same in every school $\sigma_{e}^{2}$ (homoskedasticity).

**Figure 2:**
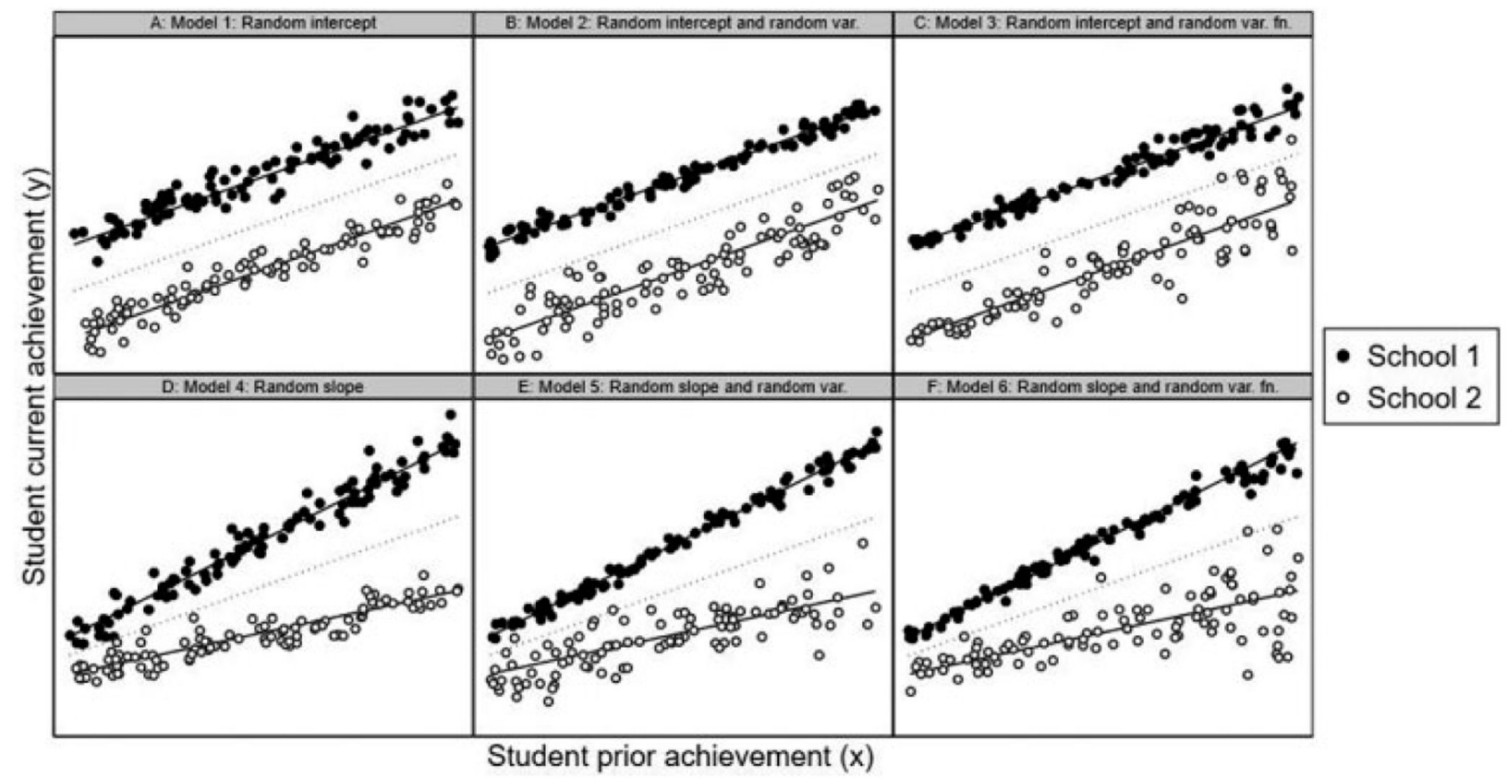
Illustration of different models using hypothetical student current and prior achievement scores data for two schools, School 1 (solid markers) and School 2 (hollow markers). Panel A: Random-intercept model. Panel B: Random-intercept model with random residual variance. Panel C: Random-intercept model with random residual variance function. Panel D: Random-slope model. Panel E: Random-slope model with random residual variance. Panel F: Random-slope model with random residual variance function.

Figure 2 illustrates the main details of this and subsequent models using hypothetical data on two schools. In each case, *y*_*ij*_ is plotted against *x*_1*ij*_. In Model 1 (Figure 2A), the two solid lines represent the school-specific relationships β_0_ + β_1_*x*_1*ij*_ *+ u*_*j*_. The dotted line depicts the average relationship between the two variables β_0_ + β_1_*x*_1*ij*_. The school lines are parallel to the average line, because in this model, only the intercept *u*_*j*_ differs between schools. The line for School 1 lies above the average line, while the line for School 2 lies below it. The vertical deviations of the school lines from the average line correspond to the school-specific *u*_*j*_. In the current example, we have *u*_1_ > 0 > *u*_2_. Thus, on average, students in School 1 are predicted to score higher compared to students with the same prior achievement in the average school, while students in School 2 are predicted to score lower. The variability in these mean deviations across all schools corresponds to $\sigma_{u}^{2}$. The vertical deviation of the student current achievement scores from their relevant school line corresponds to *e*_*ij*_. The variability in these deviations corresponds to $\sigma_{e}^{2}$. This is constant across *x*_1*ij*_ and constant across schools.

### Model 2: Random-Intercept Model With Random Residual Variance

Model 2 extends Model 1 by allowing the variance in student adjusted achievement $\sigma_{e}^{2}$ to vary across schools. We do this by specifying an MELS version of the previous model (Hedeker et al., 2008). The model can be written as

(2) $$\begin{array}{c} y_{ij}=\beta_{0}+\beta_{1}x_{1ij}+u_{j}+e_{ij},\\ \text{ln}\left(\sigma_{e,j}^{2}\right)=\alpha_{0}+v_{j},\\ (\begin{array}{c} u_{j}\\ v_{j} \end{array})\sim N\left\{(\begin{array}{c} 0\\ 0 \end{array}),\left(\begin{array}{cc} \sigma_{u}^{2} & \\ \sigma_{uv} & \sigma_{v}^{2} \end{array}\right)\right\},\\ e_{ij}\sim N\left(0,\sigma_{e,j}^{2}\right), \end{array}$$

where the second line of the equation specifies the residual variance $\sigma_{e,j}^{2}$ as a log-linear function ln(·) of a new intercept α_0_ and a new random school effect *v*_*j*_. *u*_*j*_ and *v*_*j*_ are assumed bivariate normally distributed and independent of the residuals and covariates. The variance function random intercept variance $\sigma_{v}^{2}$ measures the variation in the log of the residual variance across schools. The random intercept-slope covariance σ_*uv*_ measures how *u*_*j*_ and *v*_*j*_ covary. All other terms are defined as before. The log-linear link function ensures the resulting school-specific residual variances $\sigma_{e,j}^{2}$, and therefore, school variances of student adjusted achievement are positive (Hedeker, 2008). Figure 2B illustrates Model 2, where *v*_2_ > *v*_1_, and so, School 2 shows greater variance in their student adjusted achievements than is the case for School 1 $\sigma_{e,2}^{2}>\sigma_{e,1}^{2}$.

#### Model 3: Random-Intercept Model With Random Residual Variance Function

Recall the reason for entering student prior achievement (and potentially further student covariates) into the mean function of the model is that schools should not be held accountable for pre-existing differences in student achievement across schools at the start of the value-added period (Ballou et al., 2004; Leckie & Goldstein, 2019; Leckie & Prior, 2022; Levy et al., 2023). A similar argument applies when comparing the variance in student adjusted achievement across schools. For example, suppose the residual variance increases with increasing student prior achievement. This would suggest that schools with higher mean student prior achievement would in general be expected to show more variable student adjusted achievement than schools with lower mean student prior achievement and this is even though we have adjusted for student prior achievement in the mean function. However, following the arguments under-pinning the traditional value-added model, this should be viewed as a reflection of their school intake rather than reflecting their school policies and practices. By entering student prior achievement into the model for the variance, we adjust for this overall variance trend. Focus then shifts to how schools deviate from this overall trend.

Model 3 therefore extends Model 2 by adding student prior achievement to the residual variance function. The model is written as

(3) $$\begin{array}{c} y_{ij}=\beta_{0}+\beta_{1}x_{1ij}+u_{j}+e_{ij},\\ \ln\left(\sigma_{e,ij}^{2}\right)=\alpha_{0}+\alpha_{1}x_{1ij}+v_{j},\\ (\begin{array}{c} u_{j}\\ v_{j} \end{array})\sim N\left\{(\begin{array}{c} 0\\ 0 \end{array}),\left(\begin{array}{cc} \sigma_{u}^{2} & \\ \sigma_{uv} & \sigma_{v}^{2} \end{array}\right)\right\},\\ e_{ij}\sim N\left(0,\sigma_{e,ij}^{2}\right), \end{array}$$

where α_1_ is the residual variance function regression coefficient on *x*_1*ij*_. All other terms are defined as before. Where further student and school covariates are added to the mean function, all or a subset of these may also be added to the residual variance function. However, in order to compare school intake-adjusted values of the school variance across schools, we must now calculate the residual variance in each school at a common value of *x*_1*ij*_ such as the mean. For example, $\sigma_{e,j}^{2}=\text{exp}\left(\alpha_{0}+\alpha_{1}{\bar{x}}_{1..}+v_{j}\right)$, where ${\bar{x}}_{1..}$ denotes the mean value for *x*_1*ij*_ across all students and schools. Figure 2C illustrates Model 3, where α_1_ > 0, and so, the vertical scatter in student current achievement around each school line increases with student prior achievement in both schools and this is in addition to School 2 continuing to have greater within-school variance than school 1 (*v*_2_ > *v*_1_).

### Model 4: Random-Slope Model

Model 4 is the differential effects version (Nuttal et al., 1989; Scherer & Nilsen, 2019; Strand, 2010) of the traditional school value-added model (Model 1) and can be written as the following random-slope linear regression:

(4) $$\begin{array}{c} y_{ij}=\beta_{0}+\beta_{1}x_{1ij}+u_{0j}+u_{1j}x_{1ij}+e_{ij},\\ (\begin{array}{c} u_{0j}\\ u_{1j} \end{array})\sim N\left\{(\begin{array}{c} 0\\ 0 \end{array}),\left(\begin{array}{cc} \sigma_{u0}^{2} & \\ \sigma_{u01} & \sigma_{u1}^{2} \end{array}\right)\right\},\\ e_{ij}\sim N\left(0,\sigma_{e}^{2}\right), \end{array}$$

where *u*_0*j*_ and *u*_1*j*_ denote the random intercept and random slope effects, assumed bivariate normally distributed and independent of the residual and covariates. The random intercept variance $\sigma_{u0}^{2}$ measures the variation in school mean adjusted achievement across schools when *x*_1*ij*_ *=* 0. The random slope variance $\sigma_{u1}^{2}$ measures the variation in the slope adjustment for prior achievement across schools. The random intercept-slope covariance σ_*u*01_ measures how these two terms covary. All other terms are defined as before. Where the model includes further student covariates, their regression coefficients may also be allowed to vary across schools.

The total residual, now *u*_0*j*_ *+ u*_1*j*_*x*_1*ij*_ *+ e*_*ij*_, again measures covariate-adjusted student current achievement. However, school mean student adjusted achievement *u*_0*j*_ *+ u*_1*j*_*x*_1*ij*_ now varies not only across schools but also across students as a function of the covariate with the random slope *x*_1*ij*_. Thus, this version of the model allows schools to be potentially more or less effective for students as a function of their prior achievement.

Figure 2D illustrates Model 4, where *u*_1,1_ > *u*_1,2_, and so, School 1 shows a steeper regression line than the average line, while School 2 shows a shallower line. The school lines are given by β_0_ + β_1_*x*_1*ij*_ *+ u*_0*j*_ *+ u*_1*j*_*x*_1*ij*_. The vertical deviations of each school line from the average line correspond to *u*_0*j*_ + *u*_1*j*_*x*_1*ij*_ and so are a linear function of *x*_1*ij*_: The figure shows the school value-added score for School 1 is positive in general, but especially positive for students with high *x*_1*ij*_. In contrast, the school value-added score for School 2 is negative in general, but especially negative for students with high *x*_1*ij*_.

School mean student adjusted achievement, averaging over all students in each school, is given by $u_{0j}+u_{1j}{\bar{x}}_{1.j}$, where ${\bar{x}}_{1.j}$ denotes the average of *x*_1*ij*_ in school *j*. For the purpose of comparing schools in terms of their means, it is necessary to evaluate this quantity at common values of ${\bar{x}}_{1.j}$ for all schools. The variance in student adjusted achievement in each school (over all students) is given by $u_{1j}^{2}{\text{Var}}_{j}\left(x_{1ij}\right)+\sigma_{e}^{2}$, where Var_*j*_(*x*_1*ij*_) denotes the variance of *x*_1*ij*_ in school *j*. The first component of this expression $u_{1j}^{2}{\text{Var}}_{j}\left(x_{1ij}\right)$ captures the variance in student adjusted achievement attributable to interactions between the school effects *u*_1*j*_ and the student prior achievement *x*_1*ij*_. The magnitude of this component varies across schools. For the purpose of comparing schools in terms of their variances, it is necessary to evaluate this component at a common value of Var_*j*_(*x*_1*ij*_) for all schools, for example, the average within school variance of *x*_1*ij*_. The second component $\sigma_{e}^{2}$ is attributable to all other sources of variance in student adjusted achievement. Crucially, this continues to be assumed constant across schools (homoskedasticity). Thus, adding random slopes only partially recognizes that the variance in student adjusted achievement varies across schools.

### Model 5: Random-Slope Model With Random Residual Variance

Model 5 extends Model 4 by allowing the variance in student adjusted achievement to vary across schools. (Equally Model 5 extends Model 2 by adding a random slope in the mean function to student prior achievement.) We do this by specifying an MELS version of the previous model. The model can be written as

(5) $$\begin{array}{c} y_{ij}=\beta_{0}+\beta_{1}x_{1ij}+u_{0j}+u_{1j}x_{1ij}+e_{ij},\\ \text{ln}\left(\sigma_{e,j}^{2}\right)=\alpha_{0}+v_{j},\\ \left(\begin{array}{c} u_{0j}\\ u_{1j}\\ v_{j} \end{array}\right)\sim N\left\{\left(\begin{array}{c} 0\\ 0\\ 0 \end{array}\right),\left(\begin{array}{ccc} \sigma_{u0}^{2} & & \\ \sigma_{u01} & \sigma_{u1}^{2} & \\ \sigma_{u0v} & \sigma_{u1v} & \sigma_{v}^{2} \end{array}\right)\right\}\\ e_{ij}\sim N\left(0,\sigma_{e,j}^{2}\right), \end{array},$$

where the second line of the equation specifies the log-linear function for the residual variance (see also Model 2). The three random effects *u*_0*j*_, *u*_1*j*_, and *v*_*j*_ are assumed trivariate normally distributed and independent of the residuals and covariates. Figure 2E illustrates Model 5, where *v*_2_ > *v*_1_, and so, School 2 shows greater variance in their student adjusted achievements than is the case for School $\sigma_{e,2}^{2}>\sigma_{e,1}^{2}$ as well as a shallower slope (due to *u*_1,1_ > *u*_1,2_).

School mean student adjusted achievement (averaging over all students) is then given by $u_{0j}+u_{1j}{\bar{x}}_{1.j}$ as it was in the constant residual variance case (Model 4), and so, we will again need to evaluate this at a common value of ${\bar{x}}_{1.j}$ for all schools. The variance in student adjusted achievement in each school (over all their students) is now given by $u_{1j}^{2}{\text{Var}}_{j}\left(x_{1ij}\right)+\sigma_{e,j}^{2}$ and so differs from the constant residual variance case (Model 4) in that the last term also now varies across schools.

### Model 6: Random-Slope Model With Random Residual Variance Function

Model 6 extends Model 5 by adding student prior achievement to the residual variance function. (Equally Model 6 extends Model 3 by adding a random slope to student prior achievement.) The model is written as

(6) $$\begin{array}{c} y_{ij}=\beta_{0}+\beta_{1}x_{1ij}+u_{0j}+u_{1j}x_{1ij}+e_{ij},\\ \ln\left(\sigma_{e,ij}^{2}\right)=\alpha_{0}+\alpha_{1}x_{1ij}+v_{j},\\ \left(\begin{array}{c} u_{0j}\\ u_{1j}\\ v_{j} \end{array}\right)\sim N\left\{\left(\begin{array}{c} 0\\ 0\\ 0 \end{array}\right),\left(\begin{array}{ccc} \sigma_{u0}^{2} & & \\ \sigma_{u01} & \sigma_{u1}^{2} & \\ \sigma_{u0v} & \sigma_{u1v} & \sigma_{v}^{2} \end{array}\right)\right\},\\ e_{ij}\sim N\left(0,\sigma_{e,ij}^{2}\right), \end{array}$$

where α_1_ is the residual variance function regression coefficient on *x*_1*ij*_ (see also Model 3). All other terms are defined as before. Figure 2F illustrates Model 6, where α_1_ > 0, and so, the vertical scatter in student current achievement around each school line increases with student prior achievement and this is in addition to School 2 continuing to have a shallower slope (*u*_1,1_ > *u*_1,2_) and greater within-school variance than school 1 (*v*_2_ > *v*_1_).

As in Model 5 (and Model 4), school mean student adjusted achievement (averaging over all students) is once again given by $u_{0j}+u_{1j}{\bar{x}}_{1.j}$, while the variance in student adjusted achievement in each school (over all students) is now given by $u_{1j}^{2}{\text{Var}}_{j}\left(x_{1ij}\right)+{\text{E}}_{j}\left(\sigma_{e_{ij}}^{2}\right)$, where $E_{j}\left(\sigma_{e_{ij}}^{2}\right)$ is the mean of the student specific residual variances in school *j*. Crucially, this mean is free to vary across schools.

### Software

The traditional school value-added models (Models 1 and 4) are typically fitted via maximum likelihood estimation using conventional mixed-effects linear regression routines in standard software (R, SAS, SPSS, and Stata). However, the MELS versions of these models (Models 2, 3, 5, and 6) cannot be fitted using these routines, nor can they be fitted in specialized mixed-effects modeling packages (HLM and MLwiN). Hedeker and colleagues have developed the Mix-WILD software to fit MELS models by maximum likelihood estimation (Dzubur et al., 2020). These models can also be fitted via Markov Chain Monte Carlo (MCMC) methods in Stata and Mplus (McNeish, 2021), as well as dedicated Bayesian software such as Stan (including via the brms package in R; e.g., Parker et al., 2021), WinBUGS, OpenBUGS, and JAGS (including via the R2jags package R: e.g., Barrett et al., 2019). To support readers wishing to implement these models, we present annotated MixWILD, R, and Stata instructions and syntax and simulated data (Section S4 of the Supplemental information).

We fit all models using Stata (StataCorp, 2021). Specifically, we use the bayesmh command, which implements an adaptive Metropolis–Hastings MCMC algorithm. We use hierarchical centering reparameterizations to improve mixing. We specify vague (diffuse) normal priors for all regression coefficients and minimally informative inverse Wishart priors for the random effects variance–covariance matrices. We specify overdispersed initial values for all parameters. We fit all models with four chains, each with 5,000 burnin iterations and 10,000 monitoring iterations. We judge convergence using Gelman–Rubin convergence diagnostics (Gelman & Rubin, 1992) and trace, autocorrelation, and scatter plots. All models converged and all parameters had effective sample sizes > 400. We compare model fit using the deviance information criterion (DIC; Spiegelhalter et al., 2002). Smaller values are preferred.

## 4 Results

### Model 1: Random-Intercept Model

Model 1 (Equation 1) is the traditional school value-added model. In other words, the random-intercept model. For simplicity and because not all researchers wish to additionally include student sociodemographics (Leckie & Prior, 2022; Levy et al., 2023), we only adjust for student prior achievement in this and subsequent Models 1 through 6, but we do explore the role of further covariates in Models 7 and 8. For the purpose of comparing to subsequent models, we parameterize $\sigma_{e}^{2}$ as exp(α_0_).

Table 3 presents the results. The estimated slope coefficient on student age 11 score is ${\hat{\beta }}_{1}=0.678$, and so, a 1 *SD* difference in age 11 score is associated with a 0.678 *SD* difference in age 16 score. The estimated residual variance is ${\hat{\sigma }}_{e}^{2}=\exp(-0.870)=0.487$. The estimated total variance in student adjusted achievement is ${\hat{\sigma }}_{u}^{2}+{\hat{\sigma }}_{e}^{2}=0.487$ (and so, student age 11 scores accounts for 51% of the variation in student age 16 scores $(=100\left\{1-\left({\hat{\sigma }}_{u}^{2}+{\hat{\sigma }}_{e}^{2}\right)\right\}$; Snijders & Bosker, 2012). The estimated between-school variance in school mean adjusted achievement is ${\hat{\sigma }}_{u}^{2}=0.067$, and so, 14% of the total variation in student adjusted achievement $(=100{\hat{\sigma }}_{u}^{2}/\left({\hat{\sigma }}_{u}^{2}+{\hat{\sigma }}_{e}^{2}\right)$; Snijders & Bosker, 2012) is variation in the schools means. The between-school variance implies a 95% plausible values range (PVR) for the school means of $(-0.52,0.50)={\hat{\beta }}_{0}\pm {\text{Φ}}^{-1}(0.975)\;\sqrt{{\hat{\sigma }}_{u}^{2}}$ (where Φ ^−1^(·) denotes the inverse cumulative standard normal distribution; Raudenbush & Bryk, 2002). Thus, students in what would be deemed the most effective schools (operating at the 97.5th percentile of the distribution of all schools) are predicted to score 1.02 *SD* higher at age 16 than equivalent students in the least effective schools (operating at the 2.5th percentile). In contrast, the estimated student residual variance ${\hat{\sigma }}_{e}^{2}=\exp\left({\hat{\alpha }}_{0}\right)=0.419$, is assumed constant, naively implying the variance in student adjusted achievement is the same in every school. Plots confirm that the random effect and residual normality assumptions for this and subsequent models are reasonable (Supplemental information).

### Model 2: Random-Intercept Model With Random Residual Variance

Model 2 (Equation 2) extends the random-intercept model (Model 1, Equation 1) to allow the residual variance and therefore variance in student adjusted achievement to vary across schools. Model 2 shows a reduction in the DIC of 972 points, confirming that this variation in variances is statistically significant. The mean function parameter estimates are largely unchanged. The estimated residual variance function intercept and estimated variance of the new school random effect are ${\hat{\alpha }}_{0}=-0.881$ and ${\hat{\sigma }}_{v}^{2}=0.037$. The model-implied population-averaged school variance in student adjusted achievement is estimated as $0.422=\exp\left({\hat{\alpha }}_{0}+\frac{{\hat{\sigma }}_{v}^{2}}{2}\right)$ (Hedeker et al., 2008), which, as expected, is close to the Model 1 estimate of 0.419. The estimated population 95% PVR of school variances of student adjusted achievement is $(0.28,0.61)=\exp\left\{{\hat{\alpha }}_{0}\pm {\text{Φ}}^{-1}(0.975)\sqrt{{\hat{\sigma }}_{v}^{2}}\right\}$. This range is substantial. For example, the estimated difference in student adjusted achievement between students performing at the 97.5th and 2.5th percentile within the most variable schools ${\hat{\sigma }}_{e,j}^{2}=0.61\;$ is 3.05 *SD*, while in the least variable schools ${\hat{\sigma }}_{e,j}^{2}=0.28$, it is 2.09 *SD* (Raudenbush & Bryk, 2002).

**Figure 3:**
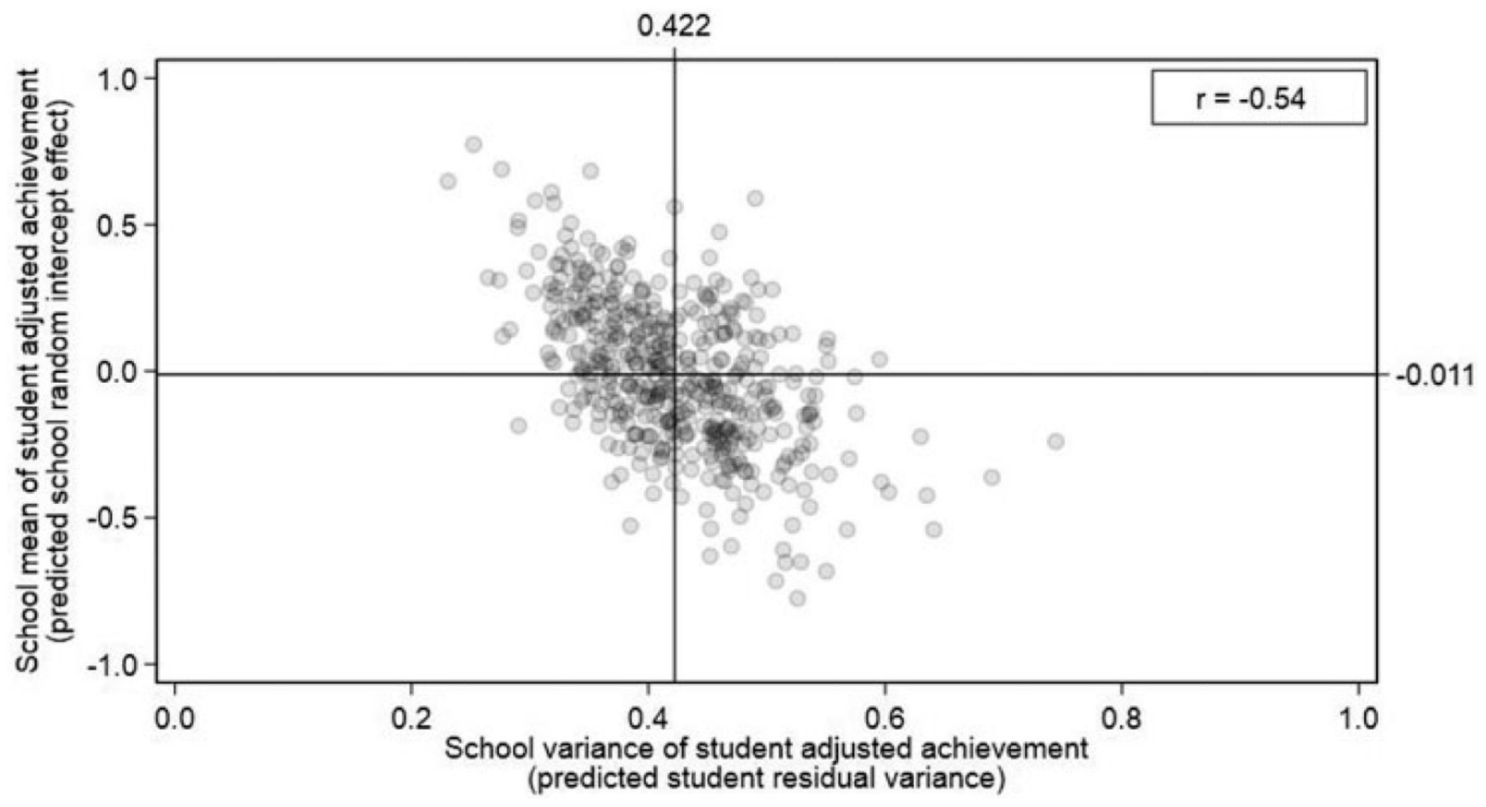
Model 2 scatterplot of school means against school variances of student adjusted achievement. London average values are shown by horizontal and vertical reference lines.

Figure 3 plots the predicted school means of student adjusted achievement *u*_*j*_ (*y*-axis) against the predicted school variances ${\hat{\sigma }}_{e,j}^{2}=\exp\left({\hat{\alpha }}_{0}+{\hat{\nu }}_{j}\right)$ (*x*-axis). The means and variances are posterior mean predictions and so have been shrunk toward their population average values as a function of their sample size (Snijders & Bosker, 2012). The London average values are illustrated by the horizontal and vertical reference lines. The figure visualizes the substantial variation in both school means and variances of student adjusted achievement described above. While the negative correlation is moderate to large *r =* −0:54, having a high school mean by no means guarantees having a low variance. Equally, there are many instances where schools show similar means but noticeably different variances.

**Figure 4:**
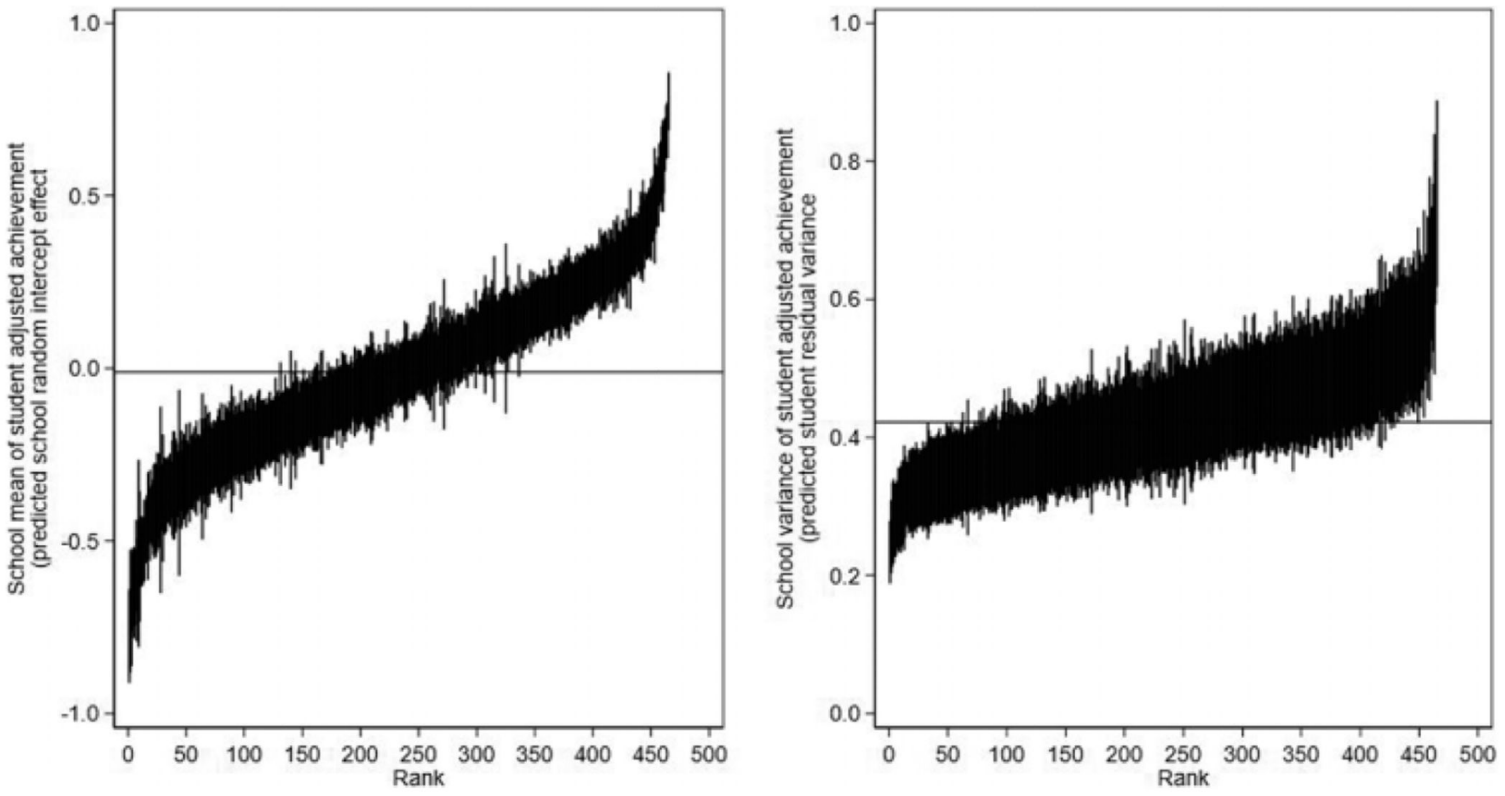
Model 2 caterpillar plots for school means (left) and school variances (right) of student adjusted achievement presented in rank order. Posterior means with 95% credible intervals.

Figure 4 presents the “caterpillar plots” of the 465 predicted school means (left panel) and school variances (right panel; Goldstein, 2011). Such plots are routinely used by researchers and accountability systems to identify schools that are significantly different from average (e.g., Prior et al., 2021). The distribution of the school variances is positively skewed, consistent with being modeled as log-normally distributed. Schools with fewer students have wider 95% credible intervals than schools with more students. Only 117 of the 465 schools (25%) can be statistically separated from the overall average in terms of their school variances compared to 320 schools (69%) when we consider the school means.

### Model 3: Random Intercept Model With Random Residual Variance Function

Model 3 (Equation 3) further extends the random-intercept model to allow the residual variance to vary not just across schools (Model 2, Equation 2), but additionally as a function of student prior achievement. Model 3 is preferred to Model 2 (ΔDIC 34), showing the residual variance significantly increases with student age 11 scores ${\hat{\alpha }}_{1}=0.029$. Thus, schools with in general higher age 11 scores are predicted to show higher variance in student adjusted achievement. However, this relationship is very weak. The estimated population 95% PVR of school intake adjusted variances of student adjusted achievement is effectively the same as in the previous model where we did not adjust for school intake, $(0.28,0.61)=\exp\left\{{\hat{\alpha }}_{0}+{\hat{\alpha }}_{1}{\bar{\bar{x}}}_{1..}\pm {\text{Φ}}^{-1}(0.975)\sqrt{{\hat{\sigma }}_{\nu }^{2}}\right\}$ where ${\bar{\bar{x}}}_{1..}=0$ denotes the London-wide average value for *x*_1*ij*_. That is, the variation in the variance in student adjusted achievement across schools is not simply explained by some schools showing in general higher age 11 scores and therefore higher variances than others.

### Model 4: Random-Slope Model

Model 4 (Equation 4) is the differential effectiveness version of the traditional school-value-added model. In other words, the random-slopes model. Recall that this model, like the traditional random-intercepts model (Model 1, Equation 1), assumes the residual variance is once again constant across all students and schools $\sigma_{e}^{2}$. As in Model 1, we parameterize $\sigma_{e}^{2}$ as exp(α_0_).

**Figure 5:**
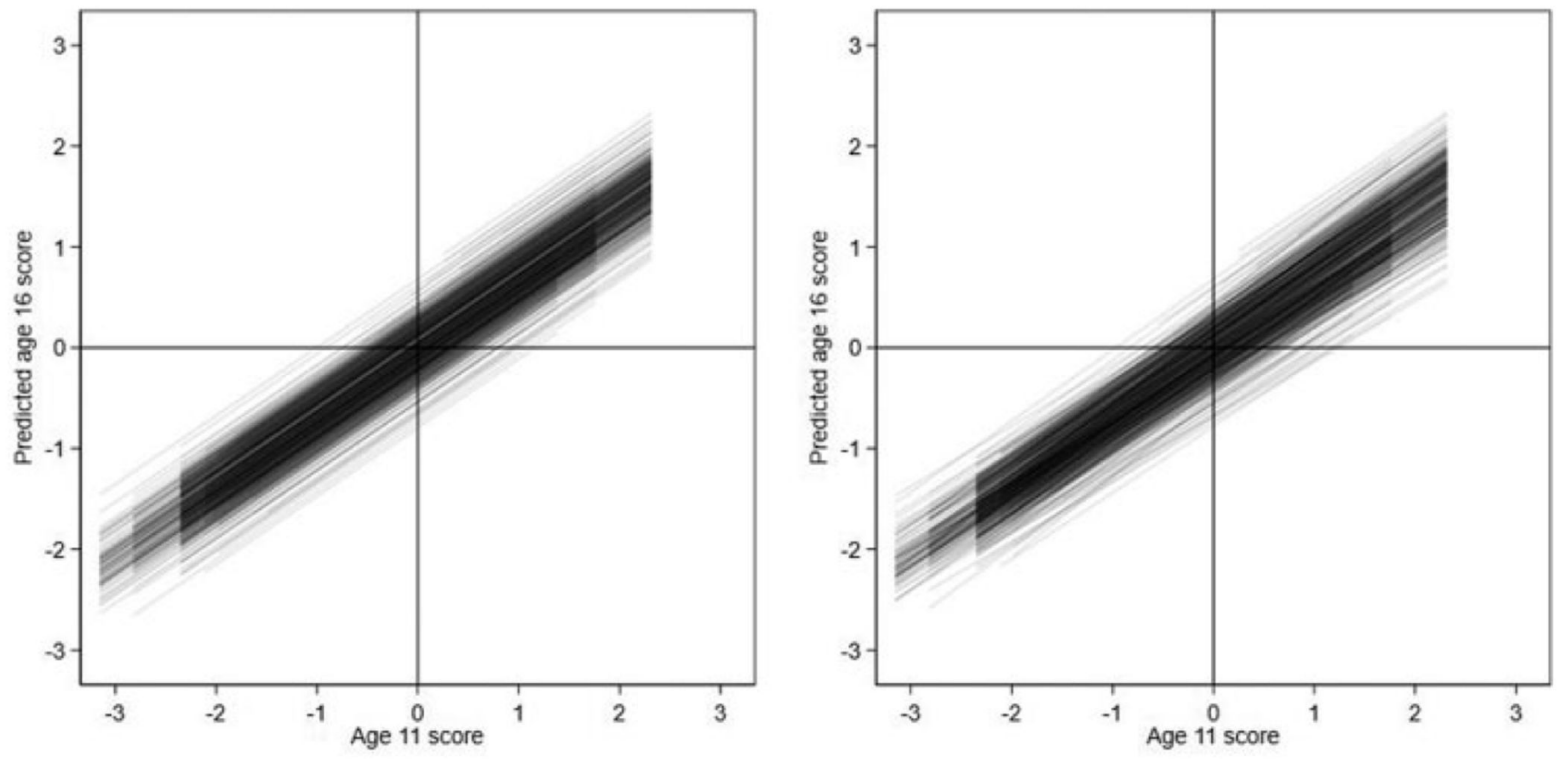
Model 1 and Model 4 school regression lines of predicted age 16 scores against age 11 scores for random-intercept model (left) and random-slope model (right).

Table 4 presents the results. Model 4 is preferred to Model 1 (ΔDIC = 281) confirming the age 11 slope varies significantly across schools. The estimated mean and variance of the age 11 slope across schools are ${\hat{\beta }}_{1}=0.675$ and ${\hat{\sigma }}_{u1}^{2}=0.004$. The latter implies an estimated 95% PVR of school slopes of $(0.55,0.80)={\hat{\beta }}_{1}\pm {\text{Φ}}^{-1}(0.975)\sqrt{{\hat{\sigma }}_{u1}^{2}}$. Figure 5 visualizes this variation for the sample schools by plotting the predicted school lines based on Model 1 (left panel) and Model 4 (right panel). The plots appear very similar, suggesting that while the random slopes are statistically significant, they are not practically significant. Indeed, moving from Model 1 to Model 4, the residual variance reduces by just 0.70%. Thus, in contrast to the literature which tends to show larger variation in school effects among low prior achievers versus high prior achievers, we find no such pattern. (Nuttal et al., 1989; Scherer & Nilsen, 2019; Strand, 2010).

### Model 5: Random-Slope Model With Random Residual Variance

Model 5 (Equation 5) extends the random-slope model (Model 4, Equation 4) to allow the residual variance to vary across schools. Thus, the move from Model 4 to 5 for the current random-slope model mirrors the move we explored from Model 1 to 2 for the earlier random-intercept versions of these models.

Model 5 allows us to quantify the relative importance of the differential school effects with respect to prior achievement as a component of the overall variance in student adjusted achievement in each school. We calculate the estimated variance for each school in our sample for a common reference distribution of students with student age 11 score variance ${\bar{s}}_{x_{1..}}^{2}=0.83$ (the mean of the sample school variances of student prior achievement). The resulting expression is ${\hat{u}}_{1j}^{2}{\bar{s}}_{x_{1..}}^{2}+{\hat{\sigma }}_{e,j}^{2}$, where ${\hat{\sigma }}_{e,j}^{2}=\exp\left({\hat{\alpha }}_{0}+{\hat{v}}_{j}\right)$ (see Section 3, Model 5). The first component ${\hat{u}}_{1j}^{2}{\bar{s}}_{x_{1..}}^{2}$ gives the variance attributable to the random slope interactions *u*_1*j*_*x*_1*ij*_. The second component ${\hat{\sigma }}_{e,j}^{2}$ captures all remaining variance. The first component is very small accounting for less than 1% of the variance in nearly all schools. In sum, the inclusion of the random slope on prior achievement has done very little to explain the variance in student adjusted achievement in each school.

### Model 6: Random-Slope Model With Random Residual Variance Function

Model 6 (Equation 6) further extends the random-slope model to allow the residual variance to vary not just across schools (Model 5, Equation 5), but additionally as a function of student prior achievement. Thus, the move from Model 5 to 6 for the current random-slope model mirrors the move we explored from Model 2 to 3 for the earlier random-intercept versions of these models. As with the sequence of random intercept models, Model 6 shows the residual variance in the random-slope model significantly increases with student age 11 scores ${\hat{\alpha }}_{1}=0.036$. However, as with the random-intercept models, this effect is slight and does little to explain the variation in school variances across schools. Given adding the random slope has little practical importance and in order to illustrate the subsequence models as simply as possible, we return to the sequence of random-intercept models.

### Model 7: Random-Intercept Model With Random Variance Function and Student Characteristics

Model 7 extends Model 3 by adding student age, gender, first language, special educational need (SEN) status, and FSM status into the mean and residual variance functions (Table 1). Adding these characteristics to the mean function implies students are now compared to other students across London who not only share the same age 11 score, but who also share the same socio-demographic characteristics. The aim is to ensure that schools do not appear more or less effective simply as a result of recruiting more or less educationally advantaged students (Leckie & Goldstein, 2019). The resulting improved accuracy of the predicted age 16 scores will lead the student adjusted achievement scores to in general reduce in absolute magnitude (and reorder) leading the overall variance in student adjusted achievement to decrease. In turn, the school means and variances of student adjusted achievement scores will also change, again in general reducing in magnitude and reordering. We then further adjust the school variances of student adjusted achievement by including the student characteristics in the student residual variance function. This ensures that if there are any London-wide relationships between the variance in student adjusted achievement and particular student characteristics, this again will not benefit or count against schools with disproportionate numbers of these students.

Table 5 presents the results. Model 7 is preferred to Model 3 (ΔDIC = 7;247) confirming the statistical importance of the student characteristics. First consider the mean function. The results show that summer born students, girls, all ethnic minority groups except mixed ethnicity students (relative to White), and students who speak English as a second language, are all predicted to score higher at age 16, than otherwise equivalent students. SEN and FSM students, in contrast, are predicted to score lower than otherwise equivalent students. These results are established and consistent with the literature (Leckie & Goldstein, 2019). What is not known is whether there are also sociodemographic differences in the variance in student adjusted achievement. The results show that, all else equal, the residual variance and therefore variance in student adjusted achievement again increases with age 11 scores but is now also shown to be higher for SEN and FSM students than for otherwise equal students. Thus, it proves harder to predict reliably the age 16 scores of these student groups relative to other student groups. In contrast, summer born students, girls, Black, and Asian students show lower variance in student adjusted achievement and therefore appear to perform in a more consistent fashion than otherwise equal student groups within schools.

**Figure 6:**
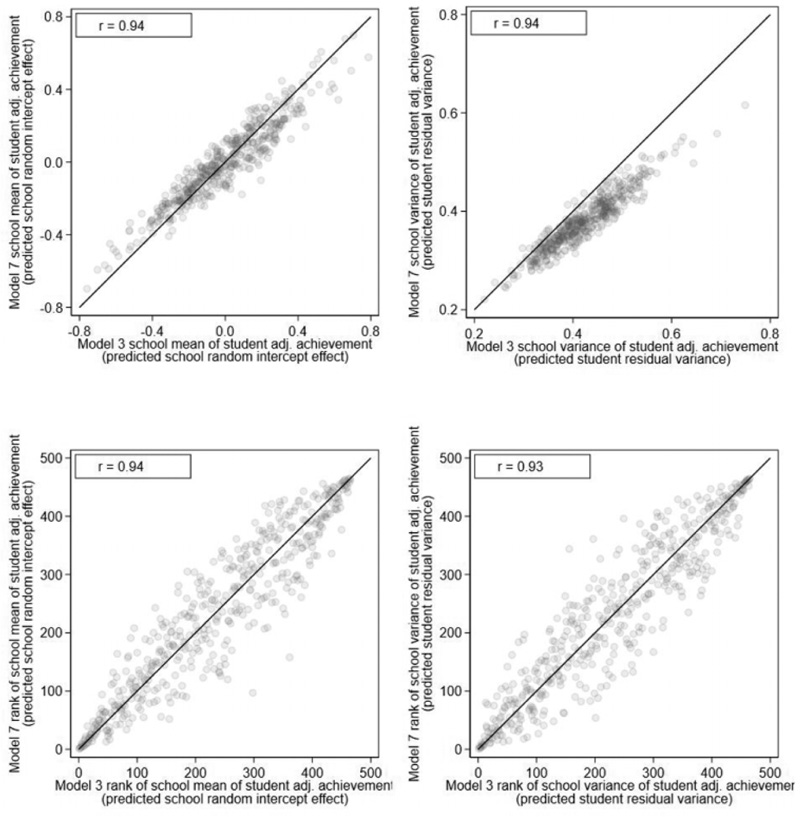
Model 7 against Model 3 scatterplots of school means of student adjusted achievement (top left), school variances of student adjusted achievement (top right), ranks of school means of student adjusted achievement (bottom left), and ranks of school variances of student adjusted achievement (bottom right).

Figure 6 presents the scatterplots of the school means and variances of student adjusted achievement based on the current model, which adjusts for student background against those based on Model 3 which ignores student background. The purpose of this figure is to explore the sensitivity of the school means and variances to the additional adjustments for student background and to therefore assess the importance of making such adjustments or not (Leckie & Prior, 2022; Levy et al., 2023). We calculate the estimated school variances in each model by plugging in the sample mean values for the covariates (Table 1) in the residual variance function, and so, ${\hat{\sigma }}_{e,j}^{2}=\exp\left({\hat{\alpha }}_{0}+{\hat{\alpha }}_{1}0.258+\ldots +{\hat{\alpha }}_{11}0.348+{\hat{v}}_{j}\right)$. The plots show both the school means and the school variances are correlated 0.94 across the two models. Thus, schools that show high mean adjusted achievement when one ignores student background nearly always still show high mean adjusted achievement after adjustment. The same applies for school variances of student adjusted achievement. However, even with such high correlations, the rank ordering of those schools whose social mix differ most markedly from the London-wide average still change considerably as shown by schools located furthest away from the 45° line in the bottom plots. Thus, the decision of whether to adjust for student background has a bearing on the manner, in which many individual schools are viewed in terms of their school variances as well as their school means.

### Model 8: Random-Intercept Model With Random Variance Function and School Characteristics

We now shift from attempting to best define and measure student adjusted achievement, and therefore the school means and variances of student adjusted achievement, to attempting to explain why some schools show higher mean student adjusted achievement and lower variance in student adjusted achievement than others. Unfortunately, we do not observe school policies and practices in our data. However, we do observe some school characteristics (Table 2). Model 8 extends Model 7 by adding school type, school admissions, school gender (mixed, boys, and girls), and school religion to the mean and residual variance functions.

The results (Table 5) for the existing mean and residual variance function regression coefficients are very similar to before and so we restrict our interpretation here to the new results. First, consider the mean function. Relative to standard school types, school mean adjusted achievement is somewhat higher in sponsored and converter academies having adjusted for the other covariates. Similarly, school mean adjusted achievement is higher in girls’ schools and religious schools, all else equal. However, the most sizable differential is related to school admissions: School mean adjusted achievement is considerably higher in grammar schools and lower in secondary modern schools relative to comprehensive schools. These results agree with the literature (Leckie & Goldstein, 2019). With respect to the residual variance function, we see new findings. School variances in student adjusted achievement tend to be lower in converter academies compared to standard school types, lower in grammar schools versus comprehensive school types, and lower in religious schools versus nonreligious schools, and this is after adjusting for London-wide relationships between the variance in student adjusted achievement and student characteristics. Thus, students in converter academies, grammar, and religious schools not only tend to show higher student adjusted achievement on average but also tend to show more consistent student adjusted achievement.

## 5 Discussion

In this article, we have argued that the focus of school value-added models should broaden to measure not just school mean differences in student adjusted achievement (student achievement beyond that predicted by student prior achievement and other student background characteristics), but school variance differences in student adjusted achievement. To study school variance differences, we have proposed extending the traditional school value-added model, a random-intercept mixed-effects linear regression of student current achievement on prior achievement and other student background characteristics, by modeling the residual variance as a log-linear function of the student covariates and a new random school effect. The school random intercept effect and random residual variance in this model measure the school mean and variance in student adjusted achievement. This model can be viewed as an application of the MELS model popular in biostatistics (Hedeker et al., 2008). It is, however, important to reiterate that the school value-added models and their respective predicted school effects should be viewed as descriptive rather than causal since these models do not address the complex selection into schools processes that will be in play in many school systems.

We have illustrated this extended school value-added model with an application to schools in London. In response to our research question: Our results suggest meaningful differences in the variance in student adjusted achievement across schools. We also find a moderate to large negative association between the school mean and variance in student adjusted achievement. Thus, schools that show the highest mean student adjusted achievement also tend to be the schools that show the lowest variance in student adjusted achievement. One process by which school variance differences may arise is if there is a London-wide negative relationship between the variance in student adjusted achievement and student prior achievement. We adjusted for this by entering student prior achievement into the residual variance function. A second process by which school variance differences may arise is via interaction effects between the different school policies and practices envisaged to be represented by the school random intercept effect and observed and unobserved student characteristics. Previous research has studied this via entering a school random slope on student prior achievement and this showed schools to be differentially effective for students with low, middle, and high prior achievement. In our application, however, these school-by-student prior achievement interactions are small and explain little of the variation in school variances between schools. We then turned our attention to entering student characteristics into the model, both in the mean and residual variance functions, to better measure student adjusted achievement. In terms of new results, we find that FSM and SEN students show greater variance in student adjusted achievement and therefore less predictable age 16 scores than otherwise equal students. The resulting predicted school means and variances of student adjusted achievement, however, are similar to those based on the model, which only adjusts for student prior achievement. Nevertheless, schools whose sociodemographic student mix differ most from the average school still move up and down the London-wide rankings considerably, demonstrating the importance of adjusting for student background at least for some schools (Leckie & Goldstein, 2019; Leckie & Prior, 2022; Levy et al., 2023). Finally, we shifted our emphasis from measuring school means and variances of student adjusted achievement to seeking to explain them. We find converter academies and grammar schools tend to show lower variances in student adjusted achievement than other school types. Importantly, here too we adjusted for any overall relationship between the variance in student adjusted achievement and student prior achievement and background characteristics, and so, these differences in school variances lie beyond this simple explanation.

Future studies might seek to identify whether school variance differences can be predicted by specific school policies and practices. It will also be interesting and important to explore the role of school composition covariates, such as the school mean and school *SD* of the student prior achievement (Raudenbush & Bryk, 2002). One issue that such studies should bear in mind is that some student current achievement measures may exhibit floor or ceiling effects. Where these are pronounced, they may bias the model parameters relative to fitting models to measures without such effects. Tobit versions of the models might be considered to address this issue (Lu, 2018). Another issue is sample-size requirements. In general, we found that the residual variance function regression coefficients and predicted school effects were less precisely estimated than their analogous quantities in the mean function. This suggests that larger sample sizes are needed for these models than traditionally used for school value-added studies. Future studies might therefore use power calculations to guide such decisions (Walters et al., 2018).

More generally, however, expanding the focus of school value-added models to consider schools effects on the variance in student achievement raises value judgements and interpretational challenges that future work will need to engage with. Fundamentally, it is not clear how positively or negatively higher or lower variances should be viewed in general. Similarly, where a given school policy or practice is identified as driving school differences in variance via differential effects on students as a function of their observed and unobserved characteristics, it will not typically be clear what the optimal degree of differential impact might be. Even if it is decided that higher variance should be interpreted in a particular way, faced now with two summaries of school effects on student learning (mean and variance effects), researchers and school accountability systems must make further value judgments as to how to best combine them into any overall summary of school effectiveness for the purpose of making overall inferences, judgements and decisions about schools (Prior, Goldstein, et al., 2021). Crucially, it is only by extending the school value-added model to allow for school effects on the variance in student adjusted achievement that such debates are made possible. The extension we have presented paves the way for new substantive research into the reasons behind differences in variability and therefore how best such differences should be interpreted and addressed.

The school value-added model presented here can be further extended in various ways beyond simply adding further covariates and random slopes suggesting avenues for new methodological research. First, in the school effectiveness literature, there is interest in studying the consistency of school effects across academic subjects (Goldstein, 1997; Reynolds et al., 2014; Teddlie & Reynolds, 2000). We can further develop the school value-added model to study this phenomenon with respect to the school variance in student adjusted achievement. Essentially, we would fit a multivariate response version of this model for multiple student achievement scores (Kapur et al., 2015; Leckie, 2018; Pugach et al., 2014). The model would have multiple residual variance functions, one for each academic subject. We can then study the correlations of the school means and variances of student adjusted achievement across subjects. Second, the same multivariate response version of the model can be used to study the stability of school effects over time. Here, we would fit a multivariate response model to a single achievement score, but for multiple student cohorts (Leckie & Goldstein, 2009). Third, we could include a random slope in the residual variance function (Goldstein et al., 2018; McNeish, 2021) to study whether schools exacerbate or mitigate any overall relationship between the variance in student adjusted achievement and student prior achievement. Fourth, while we have flexibly modeled the residual variance, we have not modeled the random intercept variance (the random slope model relaxed this, but in a rather specific way). It is also possible to model the random intercept variance as a log-linear function of school covariates (Hedeker et al, 2008). For example, the variability of school mean adjusted achievement scores across schools may appear greater for some school groups than others, and this could then be tested by introducing the school group variable as a covariate in this second variance function. Fifth, we can expand the model to three levels to incorporate an additional random effect into the mean and residual variance functions relating to, for example, school district and thereby study school district differences in the mean and variance in student adjusted achievement. This then raises the possibility of entering school district random effects into the school random intercept variance function since school mean adjusted achievement might vary more in some school districts than in others, and so with this extension, we can potentially study differential school-level inequalities in the education system by school district (Leckie et al., 2012; Leckie and Goldstein, 2015). Alternatively, teacher random effects could be introduced as a new level between the student and school level. Finally, our focus has been on shifting attention from studying school mean of student adjusted achievement to additionally focusing on the variance in student adjusted achievement. In future work, it would be interesting to explore further ways the distribution of student adjusted achievement might vary across schools, for example, with respect to skewness.

## Supplementary Material

Supplementary Materials 1-4

## Figures and Tables

**Table 1:** Summary Statistics for the Student Characteristics

	*N*	%
Age		
Not summer born	52,957	74.3
Summer born	18,364	25.8
Gender		
Boy	35,338	49.6
Girl	35,983	50.5
Ethnicity		
White	28,070	39.4
Black	15,633	21.9
Asian	14,987	21.0
Chinese	447	0.6
Mixed	5,795	8.1
Other	6,389	9.0
Language		
English	42,789	60.0
Not English	28,532	40.0
Special educational need (SEN)		
Not SEN	61,189	85.8
SEN	10,132	14.2
Free school meal (FSM)		
Not FSM	46,500	65.2
FSM	24,821	34.8

*Note. n =* 71; 321.

**Table 2:** Summary Statistics for the School Characteristics

	*n*	*%*
Type		
Standard	151	32.5
Sponsored academy	93	20.0
Converter academy	184	39.6
Other	37	8.0
Admissions		
Comprehensive	425	91.4
Grammar	19	4.1
Secondary modern	21	4.5
School gender		
Mixed	340	73.1
Boys	50	10.8
Girls	75	16.1
Religious		
No	349	75.1
Yes	116	25.0

*Note. n* = 465.

**Table 3:** Results for the Random-Intercept Models Adjusting Only for Student Prior Achievement

		Model 1			Model 2			Model 3	
		Est.	*SE*		Est.	*SE*		Est.	*SE*
		Mean Function							
β_0_	Intercept	−.011	.012		−.011	0.013		−.011	.012
β_1_	Age 11 score	.678	.003		.679	0.003		.679	.003
$\sigma_{u}^{2}$	School intercept effect variance	.067	.005		.067	0.005		.067	.005
		Residual Variance Function							
α_0_	Intercept	−.870	.005		−.881	0.010		−.881	.011
α_1_	Age 11 score							.029	.006
$\sigma_{v}^{2}$	School intercept effect variance				.037	0.003		.040	.004
		Assoc. Between Mean and Var. Fn. Random Effects							
ρ*_uv_*	Intercept effects correlation				−.472	0.048		−.484	.047
		Fit Statistics							
	DIC	140,803			139,831			139,796	

**Table 4:** Results for the Random-Slope Models Adjusting Only for Student Prior Achievement

		Model 4			Model 5			Model 6	
		Est.	*SE*		Est.	*SE*		Est.	*SE*
		Mean Function							
β_0_	Intercept	−.017	.013		−.015	.013		−.015	.013
β_1_	Age 11 score	.675	.004		.673	.004		.672	.004
$\sigma_{u0}^{2}$	School intercept effect variance	.068	.005		.069	.005		.069	.005
$\sigma_{u1}^{2}$	School slope effect variance	.004	.000		.004	.000		.004	.000
ρ_*u*__0__*u*__1_	Intercept slope effects correlation	.278	.064		.231	.066		.229	.067
		Residual Variance Function							
α_0_	Intercept	−.877	.005		−.889 .010			−.889	.011
α_1_	Age 11 score							.036	.006
$\sigma_{v}^{2}$	School intercept effect variance				.037 .003			.040	.004
		Assoc. Between Mean and Var. Fn. Random Effects							
ρ_*u*__0__*v*_	Intercept effects correlation				−.476	.048		−.494	.047
ρ_*u*__1__*v*_	Slope intercept effect correlation				−.089	.075		−.111	.076
		Fit Statistics							
	DIC	140,522			139,546			139,495	

*Note*. Est. and *SE* denote the posterior means and *SD*s of the parameter chains. DIC denotes the deviance information criterion.

**Table 5:** Results for the Random-Intercept Models Adjusting for Student Prior Achievement and Student and School Characteristics

		Model 7			Model 8	
		Est.	*SE*		Est.	*SE*
		Mean Function				
β_0_	Intercept	−.129	.012		−.235	.017
β_1_	Age 11 score	.634	.003		.632	.003
β_2_	Summer born	.045	.005		.044	.005
β_3_	Girl	.219	.005		.218	.005
β_4_	Ethnicity: Black	.015	.006		.014	.007
β_5_	Ethnicity: Asian	.152	.008		.150	.008
β_6_	Ethnicity: Chinese	.296	.028		.290	.028
β_7_	Ethnicity: Mixed	.001	.009		.000	.009
β_8_	Ethnicity: Other	.089	.010		.088	.009
β_9_	First language not English	.162	.006		.162	.006
β_10_	Special educational need (SEN)	−.276	.008		−.276	.008
β_11_	Free school meal (FSM)	−.193	.005		−.192	.005
β_12_	School type: Sponsored academy				.055	.025
β_13_	School type: Converter academy				.082	.020
β_14_	School type: Other				.023	.038
β_15_	School admissions: Grammar				.396	.049
β_16_	School admissions: Secondary modern				−.118	.045
β_17_	School gender: Boys				.053	.032
β_18_	School gender: Girls				.064	.027
β_19_	School religious				.139	.022
$\sigma_{u0}^{2}$	School intercept effect variance	.050	.004		.037	.003
		Residual Variance Function				
α_0_	Intercept	−.948	.015		−.889	.024
α_1_	Age 11 score	.077	.006		.081	.006
α_2_	Summer born	−.044	.012		−.045	.012
α_3_	Girl	−.059	.012		−.061	.012
α_4_	Ethnicity: Black	−.154	.016		−.156	.016
α_5_	Ethnicity: Asian	−.105	.018		−.106	.018
α_6_	Ethnicity: Chinese	−.088	.072		−.080	.069
α_7_	Ethnicity: Mixed	−.028	.022		−.035	.021
α_8_	Ethnicity: Other	−.014	.020		−.015	.021
α_9_	First language not English	−.002	.013		−.005	.013
α_1__0_	SEN	.204	.016		.203	.016
α_11_	FSM	.103	.012		.099	.012
α_12_	School type: Sponsored academy				.011	.028
α_13_	School type: Converter academy				−.048	.023
α_14_	School type: Other				.053	.042
α_15_	School admissions: Grammar				−.280	.052
α_16_	School admissions: Secondary modern				−.068	.044
α_17_	School gender: Boys				.002	.034
α_18_	School gender: Girls				.015	.029
α_19_	School religious				−.110	.023
$\sigma_{v}^{2}$	School intercept effect variance	.032	.003		.026	.003
		Association Between Mean and Variance Function Random Effects				
ρ_*u*__0__*v*_	Intercept effects correlation	−.409	.050		−.282	.057
		Fit Statistics				
	Deviance information criterion (DIC)	132,549			132,539	

*Note*. Est. and *SE* denote the posterior means and *SD*s of the parameter chains. Student ethnicity reference group is White. School type reference group is standard. School admissions reference group is comprehensive. School gender reference group is mixed-sex school.

## References

[R1] Aitkin M, Longford N (1986). Statistical modelling issues in school effectiveness studies. Journal of the Royal Statistical Society Series A (General).

[R2] American Statistical Association (2014). ASA Statement on Using Value-Added Models for Educational Assessment.

[R3] Amrein-Beardsley A (2014). Rethinking value-added models in education: Critical perspectives on tests and assessment-based accountability.

[R4] Amrein-Beardsley A, Holloway J (2019). Value-added models for teacher evaluation and accountability: Commonsense assumptions. Educational Policy.

[R5] Angrist J, Hull P, Pathak PA, Walters C (2021). Credible school value-added with undersubscribed school lotteries. The Review of Economics and Statistics.

[R6] Ballou D, Sanders W, Wright P (2004). Controlling for student background in value-added assessment of teachers. Journal of Educational and Behavioral Statistics.

[R7] Barrett JK, Huille R, Parker RMA, Yano Y, Griswold M (2019). Estimating the association between blood pressure variability and cardiovascular disease: An application using the ARIC Study. Statistics in Medicine.

[R8] Braun HI, Wainer H (2007). Handbook of Statistics.

[R9] Brunton-Smith I, Sturgis P, Leckie G (2017). Detecting and understanding interviewer effects on survey data by using a cross-classified mixed effects location–scale model. Journal of the Royal Statistical Society: Series A (Statistics in Society).

[R10] Brunton-Smith I, Sturgis P, Leckie G (2018). How collective is collective efficacy? The importance of consensus in judgments about community cohesion and willingness to intervene. Criminology.

[R11] Castellano KE, Ho AD (2013). A practitioner’s guide to growth models.

[R12] De Fraine B, Van Damme J, Onghena P (2002). Accountability of schools and teachers: What should be taken into account?. European Educational Research Journal.

[R13] Department for Education (2020). Secondary accountability measures: Guide for maintained secondary schools; academies, and free schools.

[R14] Department for Education (2023). National pupil database.

[R15] Dzubur E, Ponnada A, Nordgren R, Yang CH, Intille S, Dunton G, Hedeker D (2020). MixWILD: A program for examining the effects of variance and slope of time-varying variables in intensive longitudinal data. Behavior Research Methods.

[R16] Gelman A, Rubin DB (1992). Inference from iterative simulation using multiple sequences. Statistical Science.

[R17] Goldstein H (1997). Methods in school effectiveness research. School Effectiveness and School Improvement.

[R18] Goldstein H (2011). Multilevel statistical models.

[R19] Goldstein H (2020). Living by the evidence. Significance.

[R20] Goldstein H, Leckie G, Charlton C, Tilling K, Browne WJ (2018). Multilevel growth curve models that incorporate a random coefficient model for the level 1 variance function. Statistical Methods in Medical Research.

[R21] Goldstein H, Rasbash J, Yang M, Woodhouse G, Pan H, Nuttall D, Thomas S (1993). A multilevel analysis of school examination results. Oxford Review of Education.

[R22] Goldstein H, Spiegelhalter DJ (1996). League tables and their limitations: Statistical issues in comparisons of institutional performance. Journal of the Royal Statistical Society: Series A (Statistics in Society).

[R23] Hedeker D, Mermelstein RJ, Demirtas H (2008). An application of a mixed-effects location scale model for analysis of ecological momentary assessment (EMA) data. Biometrics.

[R24] Hedeker D, Mermelstein RJ, Demirtas H (2012). Modeling between-subject and within-subject variances in ecological momentary assessment data using mixed-effects location scale models. Statistics in Medicine.

[R25] Kapur K, Li X, Blood EA, Hedeker D (2015). Bayesian mixed-effects location and scale models for multivariate longitudinal outcomes: An application to ecological momentary assessment data. Statistics in Medicine.

[R26] Koretz D (2017). The testing charade: Pretending to make schools better.

[R27] Leckie G (2018). Avoiding bias when estimating the consistency and stability of value-added school effects using multilevel models. Journal of Educational and Behavioral Statistics.

[R28] Leckie G, French R, Charlton C, Browne W (2014). Modeling heterogeneous variance-covariance components in two-level models. Journal of Educational and Behavioral Statistics.

[R29] Leckie G, Goldstein H (2009). The limitations of using school league tables to inform school choice. Journal of the Royal Statistical Society: Series A (Statistics in Society).

[R30] Leckie G, Goldstein H (2015). A multilevel modelling approach to measuring changing patterns of ethnic composition and segregation among London secondary schools, 2001-2010. Journal of the Royal Statistical Society: Series A (Statistics in Society).

[R31] Leckie G, Goldstein H (2017). The evolution of school league tables in England 1992–2016: “Contextual value-added,” “expected progress” and “progress 8”. British Educational Research Journal.

[R32] Leckie G, Goldstein H (2019). The importance of adjusting for student background in school value-added models: A study of Progress 8 and school accountability in England. British Educational Research Journal.

[R33] Leckie G, Pillinger R, Jones K, Goldstein H (2012). Multilevel modelling of social segregation. Journal of Educational and Behavioral Statistics.

[R34] Leckie G, Prior L (2022). A comparison of value-added models for school accountability. School Effectiveness and School Improvement.

[R35] Levy J, Brunner M, Keller U, Fischbach A (2019). Methodological issues in value-added modeling: An international review from 26 countries. Educational Assessment, Evaluation and Accountability.

[R36] Levy J, Brunner M, Keller U, Fischbach A (2023). How sensitive are the evaluations of a school’s effectiveness to the selection of covariates in the applied value-added model?. Educational Assessment, Evaluation and Accountability.

[R37] Lu T (2018). Mixed-effects location and scale Tobit joint models for heterogeneous longitudinal data with skewness, detection limits, and measurement errors. Statistical Methods in Medical Research.

[R38] McCaffrey DF, Lockwood JR, Koretz D, Louis TA, Hamilton L (2004). Models for value-added modeling of teacher effects. Journal of Educational and Behavioral Statistics.

[R39] McNeish D (2021). Specifying location scale models for heterogeneous variances as multilevel SEMs. Organizational Research Methods.

[R40] Nordgren R, Hedeker D, Dunton G, Yang CH (2020). Extending the mixed-effects model to consider within-subject variance for ecological momentary assessment data. Statistics in Medicine.

[R41] Nuttall DL, Goldstein H, Prosser R, Rasbash J (1989). Differential school effectiveness. International Journal of Educational Research.

[R42] Organization for Economic Cooperation and Development (2008). Measuring improvements in learning outcomes: Best practices to assess the value-added of schools.

[R43] Parker RMA, Leckie G, Goldstein H, Howe LD, Heron J, Hughes AD, Phillippo DM, Tilling K (2021). Joint modeling of individual trajectories, within-individual variability, and a later outcome: Systolic blood pressure through childhood and left ventricular mass in early adulthood. American Journal of Epidemiology.

[R44] Prior L, Goldstein H, Leckie G (2021). School value-added models for multivariate academic and non-academic outcomes: Exploring implications for performance monitoring and accountability. School Effectiveness and School Improvement.

[R45] Prior L, Jerrim J, Thomson D, Leckie G (2021). A review and evaluation of secondary school accountability in England: Statistical strengths, weaknesses, and challenges for “Progress 8”. Review of Education.

[R46] Pugach O, Hedeker D, Mermelstein RJ (2014). A bivariate mixed-effects location-scale model with application to ecological momentary assessment (EMA) data. Health Services and Outcomes Research Methodology.

[R47] Rast P, Hofer SM, Sparks C (2012). Modeling individual differences in within-person variation of negative and positive affect in a mixed effects location scale model using BUGS/JAGS. Multivariate Behavioral Research.

[R48] Raudenbush SW, Bryk AS (1986). A hierarchical model for studying school effects. Sociology of Education.

[R49] Raudenbush SW, Bryk AS (2002). Hierarchical linear models: Applications and data analysis methods.

[R50] Raudenbush SW, Willms JD (1995). The estimation of school effects. Journal of Educational and Behavioral Statistics.

[R51] Reardon SF, Raudenbush SW (2009). Assumptions of value-added models for estimating school effects. Education Finance and Policy.

[R52] Reynolds D, Sammons P, De Fraine B, Van Damme J, Townsend T, Teddlie C, Stringfield S (2014). Educational effectiveness research (EER): A state-of-the-art review. School Effectiveness and School Improvement.

[R53] Rubin DB, Stuart EA, Zanutto EL (2004). A potential outcomes view of value-added assessment in education. Journal of Educational and Behavioral Statistics.

[R54] Scherer R, Nilsen T (2019). Closing the gaps? Differential effectiveness and accountability as a road to school improvement. School Effectiveness and School Improvement.

[R55] Snijders TAB, Bosker RJ (2012). Multilevel analysis: An introduction to basic and advanced multilevel modeling.

[R56] Spiegelhalter DJ, Best NG, Carlin BP, Van Der Linde A (2002). Bayesian measures of model complexity and fit. Journal of the Royal Statistical Society, Series B.

[R57] Strand S (2010). Do some schools narrow the gap? Differential school effectiveness by ethnicity, gender, poverty, and prior achievement. School Effectiveness and School Improvement.

[R58] StataCorp (2021). Stata 17 Bayesian analysis reference manual.

[R59] Teddlie C, Reynolds D (2000). The international handbook of school effectiveness research.

[R60] Thomas S, Mortimore P (1996). Comparison of value-added models for secondary-school effectiveness. Research Papers in Education.

[R61] Timmermans AC, Thomas SM (2015). The impact of student composition on schools’ value-added performance: A comparison of seven empirical studies. School Effectiveness and School Improvement.

[R62] Wainer H (2004). Introduction to a special issue of the journal of educational and behavioral statistics on value-added assessment. Journal of Educational and Behavioral Statistics.

[R63] Walters RW, Hoffman L, Templin J (2018). The power to detect and predict individual differences in intra-individual variability using the mixed-effects location-scale model. Multivariate Behavioral Research.

