# Supplementary material for: Mixed-Effects Location Scale Models for Joint Modeling School Value-Added Effects on the Mean and Variance of Student Achievement
Source: J Educ Behav Stat. Author manuscript; Available in PMC 2025 Apr 8. (PMC7617570; doi:10.3102/10769986231210808)
Supplement: Supplementary Materials 1-4 [file EMS203641-supplement-Supplementary_Materials_1_4.zip › sj-docx-1-jeb-10.3102_10769986231210808.docx]

**Mixed-Effects Location Scale Models for Joint Modeling School Value-Added Effects on the Mean and Variance of Student Achievement**

# Supplementary File

# S1. Supplementary Figures


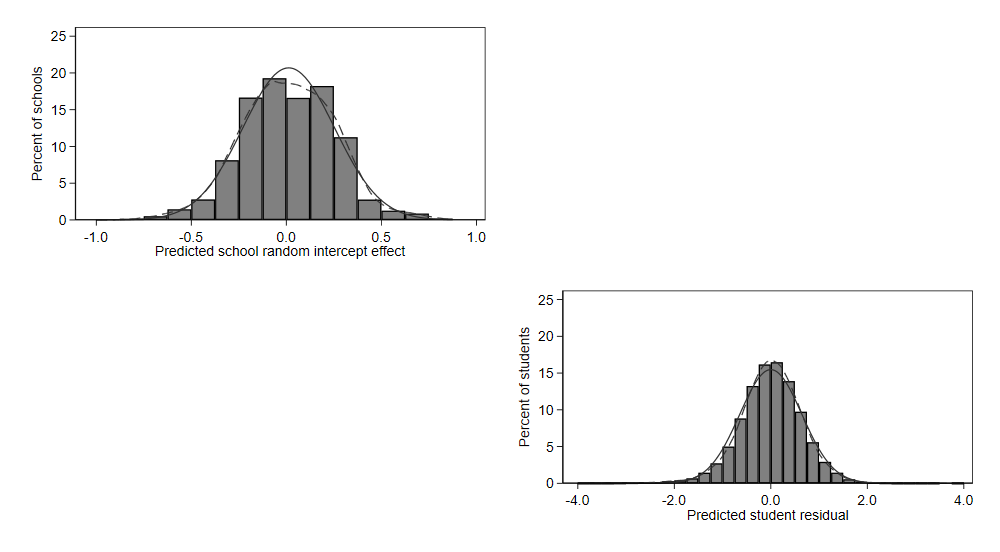


### FIGURE S1. *Model 1 histograms of predicted school random intercept effects (top left) and student residuals (bottom right), each with superimposed normal curves (solid) and kernel density curves (dashed).*


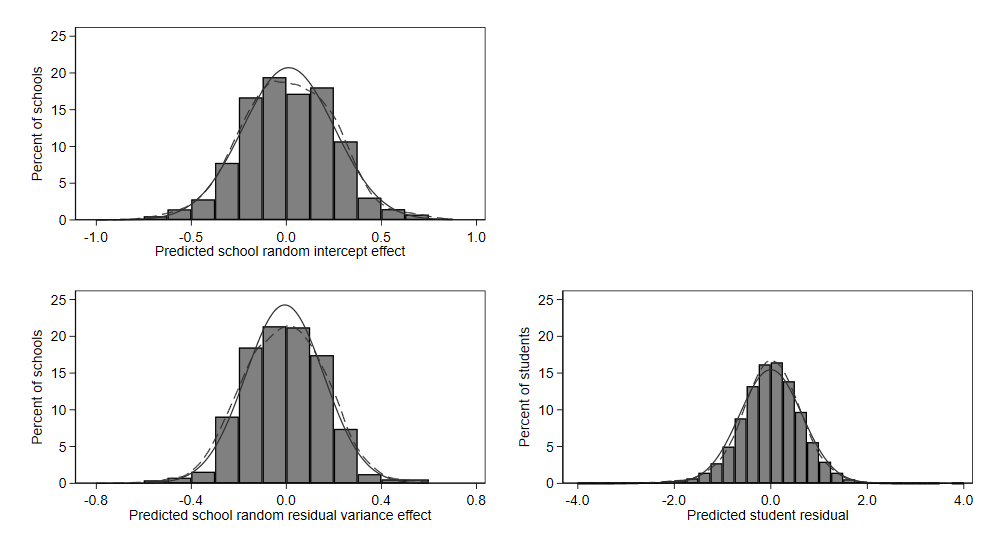


### FIGURE S2. *Model 2 histograms of predicted school random intercept effects (top left), school random residual variance effects (bottom left), and student residuals (bottom right), each with superimposed normal curves (solid) and kernel density curves (dashed).*

###
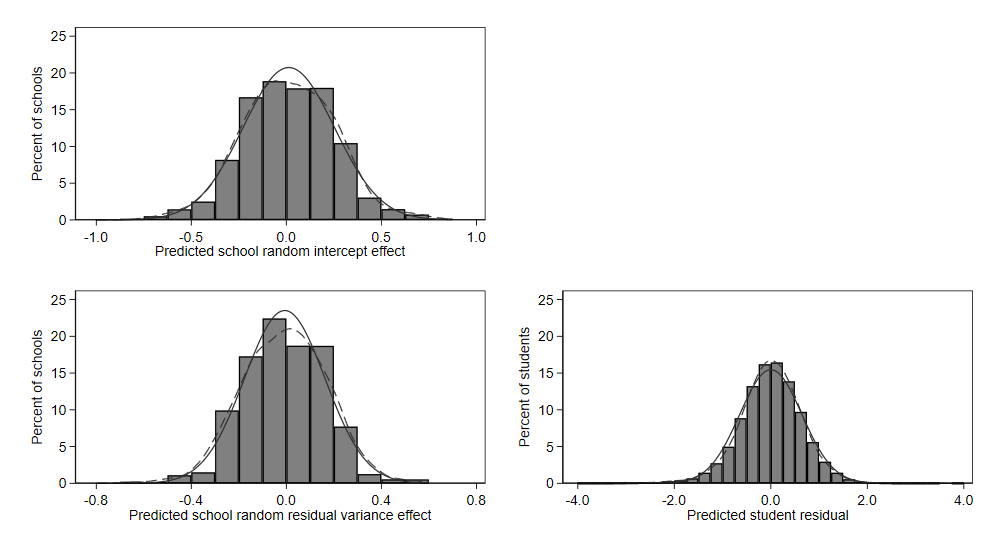
FIGURE S3. *Model 3 histograms of predicted school random intercept effects (top left), school random residual variance effects (bottom left), and student residuals (bottom right), each with superimposed normal curves (solid) and kernel density curves (dashed).*

###
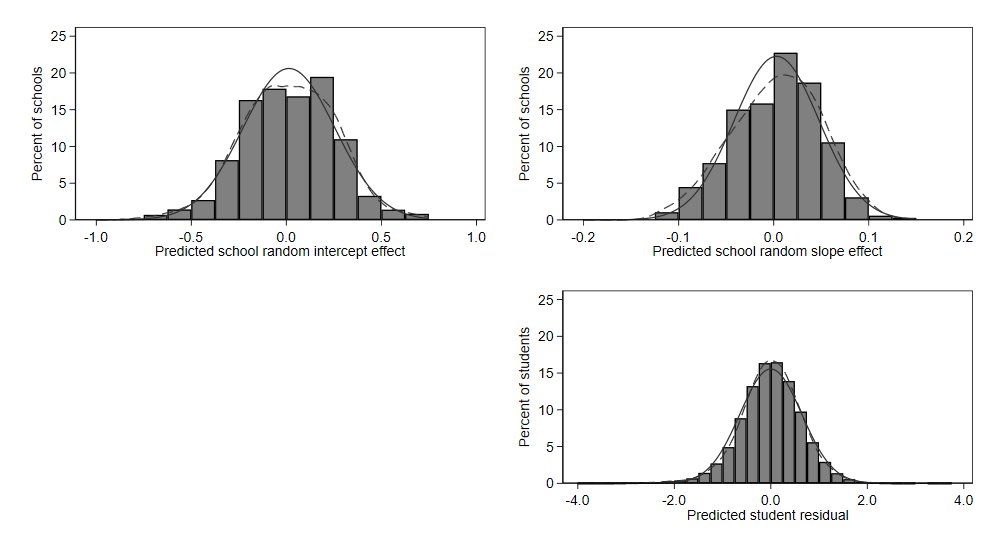
FIGURE S4. *Model 4 histograms of predicted school random intercept effects (top left), school random slope effects (top right), and student residuals (bottom right), each with superimposed normal curves (solid) and kernel density curves (dashed).*

###
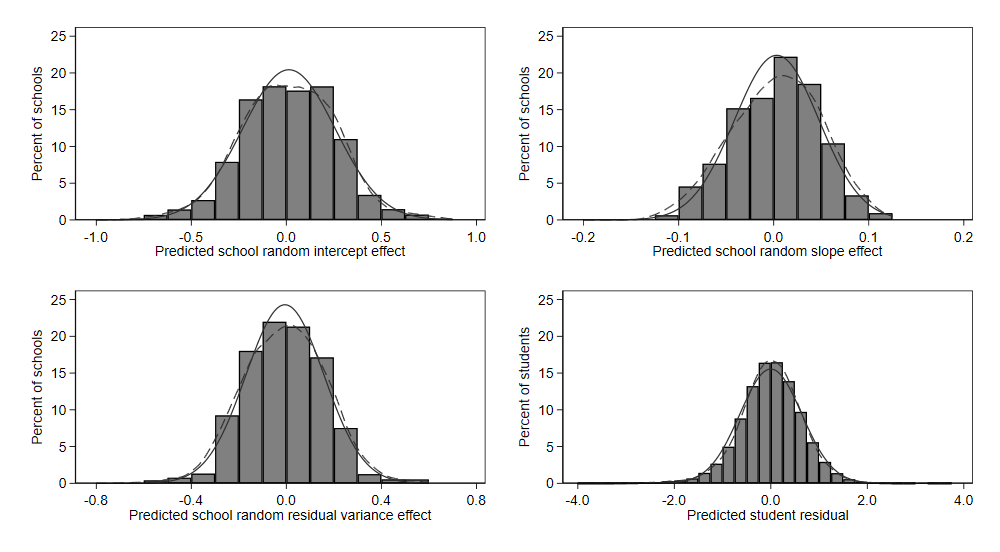
FIGURE S5. *Model 5 histograms of predicted school random intercept effects (top left), school random slope effects (top right), school residual variance effects (bottom left), and student residuals (bottom right), each with superimposed normal curves (solid) and kernel density curves (dashed).*


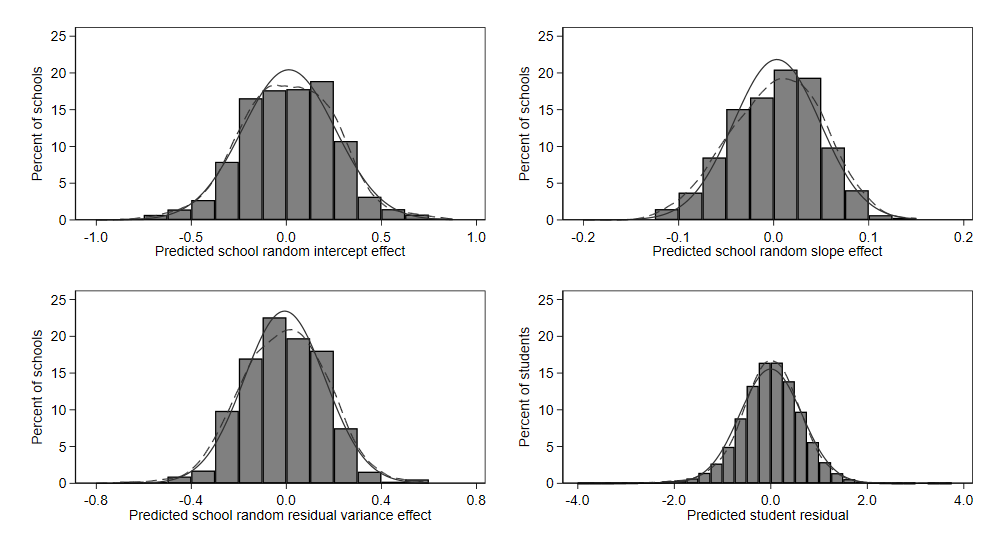


### FIGURE S6. *Model 6 histograms of predicted school random intercept effects (top left), school random slope effects (top right), school residual variance effects (bottom left), and student residuals (bottom right), each with superimposed normal curves (solid) and kernel density curves (dashed).*

###
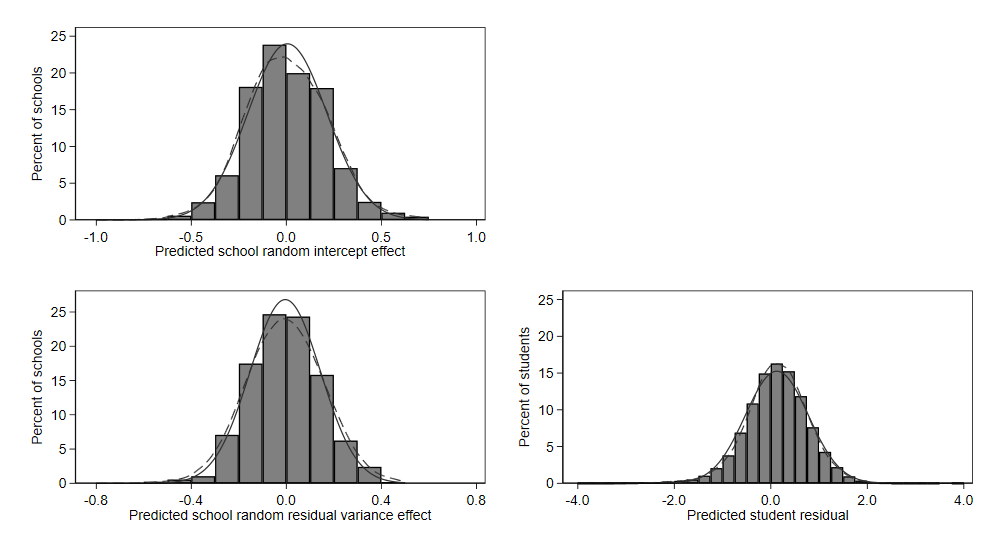
FIGURE S7. *Model 7 histograms of predicted school random intercept effects (top left), school random residual variance effects (bottom left), and student residuals (bottom right), each with superimposed normal curves (solid) and kernel density curves (dashed).*

###
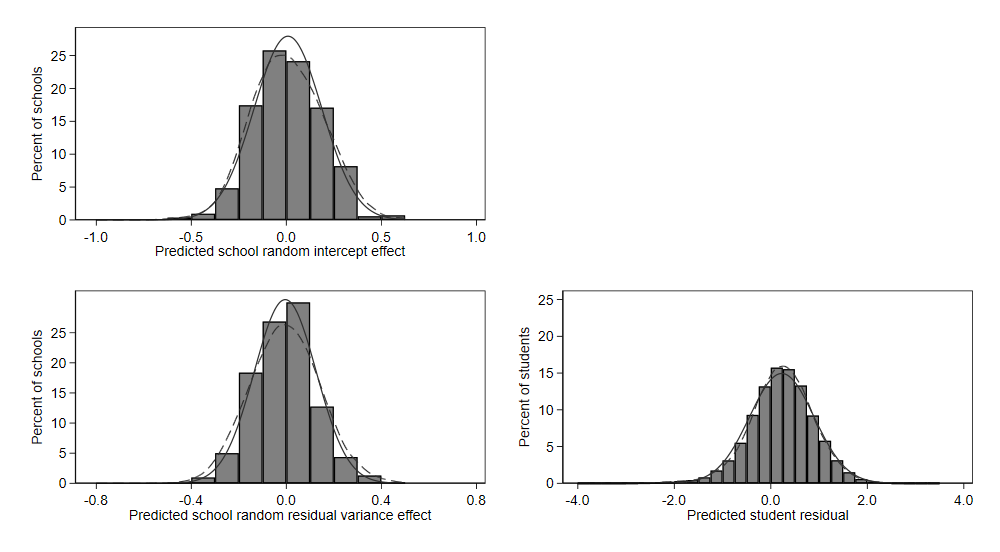
FIGURE S8. *Model 8 histograms of predicted school random intercept effects (top left), school random residual variance effects (bottom left), and student residuals (bottom right), each with superimposed normal curves (solid) and kernel density curves (dashed).*

# S2. Stata, R and MixWILD Software Instructions and Simulated Data for Fitting the Models

In this section, we describe Stata and R syntax to fit the models explored in this article by MCMC methods and MixWILD point-and-click instructions to fit these models by maximum likelihood estimation. To support readers, we provide script files and data to replicate the presented analysis.

## Example Model

For simplicity, we focus on the two-level random-intercept model with a random residual variance function presented in Section 3. To illustrate the syntax as simply as possible, we consider a version of this model with only one student characteristic (student prior achievement). This model can be written as

$y_{ij}=\beta_{0}+\beta_{1}x_{ij}+u_{j}+e_{ij}$ (S1)

$$\ln\left( \sigma_{e,ij}^{2} \right)=\alpha_{0}+\alpha_{1}x_{ij}+v_{j}$$

$$\left( \begin{matrix} u_{j} \\ v_{j} \end{matrix} \right) \sim N\left\{ \left( \begin{matrix} 0 \\ 0 \end{matrix} \right),\left( \begin{matrix} \sigma_{u}^{2} & \\ \sigma_{uv} & \sigma_{v}^{2} \end{matrix} \right) \right\}$$

$$e_{ij}\sim N\left( 0,\sigma_{e,ij}^{2} \right)$$

This model is the same as Model 3 presented in the article. Model 1 presented in the article can be viewed as a constrained version of this model (where the residual variance is assumed constant across all students and schools). Models 2, 5, and 6 vary in the covariates included in both the mean and residual variance functions. Model 4 is a random-slope version of this model (where a random slope is added to prior achievement).

## Simulated Data

As we cannot share the data analyzed in the article, we analyze here simulated data where we use the above model as the data generating model. We simulate a single dataset with 100 schools and 25 students per school. We simulate $x_{ij}$ as standard normal variate with intraclass correlation of 0.2. We specify the true parameter values as $\beta_{0}=0$, $\beta_{1}=0.7$, $\sigma_{u}^{2}=0.05$, $\alpha_{0}=-0.8$, $\alpha_{1}=0.05$, $\sigma_{v}^{2}=0.05$, $\sigma_{uv}=0.025$. The resulting data can be found in the files data.dta and data.csv.

## Stata: The bayesmh Command

We focus on the bayesmh Stata command (StataCorp, 2021). The bayesmh command implements an adaptive Metropolis-Hastings MCMC algorithm. We present the simplest possible syntax noting that mixing can be improved via model reparameterization (e.g., hierarchical centering) and by specifying various estimation options (initial values, blocking) and we encourage readers to consult the comprehensive documentation for further details.

The syntax to specify and fit this model is as follows.

. bayesmh y x U[school], ///

likelihood(normal(exp({lnsigma2e:x,xb} + {V[school]}))) ///

prior({y:}, normal(0, 10000)) ///

prior({lnsigma2e:}, normal(0, 10000)) ///

prior({U} {V}, mvnormal(2, 0, 0, {SIGMAUV, matrix})) ///

prior({SIGMAUV, matrix}, iwishart(2, 3, S))

Line 1 of the syntax specifies the mean function. Line 2 specifies the normal response distribution and the residual variance function. The intercept is included in both functions by default. Lines 3 and 4 specify diffuse normal priors for the regression coefficients in each function with means of 0 and variances of 10000. Line 5 specifies the random effects to be bivariate normally distributed with zero means and a constant covariance matrix. Line 6 specifies a minimally informative inverse Wishart distribution for this covariance matrix (where S is pre-specified matrix such as an identity matrix).

The associated model output is as follows

Burn-in 2500 aaaaaaaaa1000aaaaaaaaa2000aaaaa done

Simulation 10000 .........1000.........2000.........3000.........4000.........5000.........6000......

> ...7000.........8000.........9000.........10000 done

Model summary

------------------------------------------------------------------------------

Likelihood:

y ~ normal(xb_y,exp({lnsigma2e:x,xb} + {V[school]}))

Prior:

{y:x _cons} ~ normal(0,10000) (1)

Hyperpriors:

{lnsigma2e:x _cons} ~ normal(0,10000)

{U[school] V[school]} ~ mvnormal(2,0,0,{SIGMAUV,m})

{SIGMAUV,m} ~ iwishart(2,3,S)

------------------------------------------------------------------------------

(1) Parameters are elements of the linear form xb_y.

Bayesian normal regression MCMC iterations = 12,500

Random-walk Metropolis–Hastings sampling Burn-in = 2,500

MCMC sample size = 10,000

Number of obs = 2,500

Acceptance rate = .196

Efficiency: min = .002048

avg = .01894

Log marginal-likelihood max = .04904

------------------------------------------------------------------------------

| Equal-tailed

| Mean Std. dev. MCSE Median [95% cred. interval]

-------------+----------------------------------------------------------------

y |

x | .7005516 .0142504 .000858 .7005228 .6731723 .7301972

_cons | -.0282751 .0220327 .001753 -.0289124 -.0706135 .0183987

-------------+----------------------------------------------------------------

lnsigma2e |

x | .0520455 .0299735 .001694 .0520977 -.0059208 .1090707

_cons | -.7796784 .0347738 .00157 -.7794015 -.8489381 -.7097778

-------------+----------------------------------------------------------------

SIGMAUV_1_1 | .0340151 .0068176 .001046 .0338658 .0200429 .0490111

SIGMAUV_2_1 | .0137237 .0077867 .001553 .0138888 -.0011668 .0284148

SIGMAUV_2_2 | .0431452 .0209756 .004636 .0395217 .0092352 .0902076

------------------------------------------------------------------------------

Note: There is a high autocorrelation after 500 lags.

Note: Adaptation tolerance is not met in at least one of the blocks.

The command ran one chain with 2500 burn-in iterations and 10000 monitoring iterations. The reader should note the warning messages at the end of the output. As noted above, mixing can be improved via model reparameterization and by specifying various estimation options. The results presented in tabular form are as follows

|  | True value | Est. | SE |
| --- | --- | --- | --- |
| $\beta_{0}$ | 0.000 | -0.028 | 0.022 |
| $\beta_{1}$ | 0.700 | 0.701 | 0.014 |
| $\sigma_{u}^{2}$ | 0.050 | 0.034 | 0.007 |
| $\alpha_{0}$ | -0.800 | -0.780 | 0.035 |
| $\alpha_{1}$ | 0.050 | 0.052 | 0.030 |
| $\sigma_{v}^{2}$ | 0.050 | 0.043 | 0.021 |
| $\sigma_{uv}$ | 0.025 | 0.014 | 0.008 |

The parameter estimates are similar to their true values and to those provided by brms in R and MixWILD (see below).

## R: The brms Package

We focus on the brm function of the brms R package (Bürkner, 2017, 2018). The brms package calls the Stan software (Stan Development Team, 2021) which implements Hamiltonian Monte Carlo (HMC) and no-U-turn samplers (NUTS). We present the simplest possible syntax noting that mixing can be improved via model reparameterization and by specifying various estimation options and we encourage readers to consult the comprehensive documentation for further details.

The brms package specifies model S1 using the following alternative parameterization.

$y_{ij}=\beta_{0}+\beta_{1}x_{ij}+u_{j}+e_{ij}$ (S2)

$$\ln\left( \sigma_{e,ij} \right)=\alpha_{0}^{'}+\alpha_{1}^{'}x_{ij}+v_{j}^{'}$$

$$\left( \begin{matrix} u_{j} \\ v_{j}^{'} \end{matrix} \right) \sim N\left\{ \left( \begin{matrix} 0 \\ 0 \end{matrix} \right),\left( \begin{matrix} \sigma_{u}^{2} & \\ \sigma_{uv^{'}} & \sigma_{v^{'}}^{2} \end{matrix} \right) \right\}$$

Thus, the residual variance function is now specified in terms of the residual SD. Fortunately, the parameter and random effect values of the original parameterization can be easily recovered as follows $\alpha_{0}=2\alpha_{0}^{'}$, $\alpha_{1}=2\alpha_{1}^{'}$, $v_{j}=2v_{j}^{'},\sigma_{v}^{2}=4\sigma_{v^{'}}^{2}$.

The syntax to specify and fit this model is as follows

brm(bf(y ~ 1 + x + (1 |s| school),

sigma ~ 1 + x + (1 |s| school)),

data = mydata,

family = gaussian()

)

where for further simplicity we use the default priors for all model parameters and random effects. These include improper flat priors for the regression coefficients, half student-t priors with three degrees of freedom for the random effect standard deviations, and the LKJcorr prior for random effect correlation matrix (Bürkner, 2017).

Line 1 of the brm function syntax specifies the mean function. Line 2 specifies the residual variance function parameterized in terms of the residual SD rather than the residual variance. In these two lines, the “|s|” is used to indicate that the mean function and residual variance function random effects are part of the same set and will therefore by default be allowed to correlate. Line 3 specifies the data frame. Line 4 specifies the normal response distribution.

The associated model output is as follows.

Compiling Stan program...

Start sampling

SAMPLING FOR MODEL '93a90408567ae7343eea598de7d7e540' NOW (CHAIN 1).

Chain 1:

Chain 1: Gradient evaluation took 0.005 seconds

Chain 1: 1000 transitions using 10 leapfrog steps per transition would take 50 seconds.

Chain 1: Adjust your expectations accordingly!

Chain 1:

Chain 1:

Chain 1: Iteration: 1 / 2000 [ 0%] (Warmup)

Chain 1: Iteration: 200 / 2000 [ 10%] (Warmup)

Chain 1: Iteration: 400 / 2000 [ 20%] (Warmup)

Chain 1: Iteration: 600 / 2000 [ 30%] (Warmup)

Chain 1: Iteration: 800 / 2000 [ 40%] (Warmup)

Chain 1: Iteration: 1000 / 2000 [ 50%] (Warmup)

Chain 1: Iteration: 1001 / 2000 [ 50%] (Sampling)

Chain 1: Iteration: 1200 / 2000 [ 60%] (Sampling)

Chain 1: Iteration: 1400 / 2000 [ 70%] (Sampling)

Chain 1: Iteration: 1600 / 2000 [ 80%] (Sampling)

Chain 1: Iteration: 1800 / 2000 [ 90%] (Sampling)

Chain 1: Iteration: 2000 / 2000 [100%] (Sampling)

Chain 1:

Chain 1: Elapsed Time: 41.769 seconds (Warm-up)

Chain 1: 19.914 seconds (Sampling)

Chain 1: 61.683 seconds (Total)

Chain 1:

SAMPLING FOR MODEL '93a90408567ae7343eea598de7d7e540' NOW (CHAIN 2).

Chain 2:

Chain 2: Gradient evaluation took 0.001 seconds

Chain 2: 1000 transitions using 10 leapfrog steps per transition would take 10 seconds.

Chain 2: Adjust your expectations accordingly!

Chain 2:

Chain 2:

Chain 2: Iteration: 1 / 2000 [ 0%] (Warmup)

Chain 2: Iteration: 200 / 2000 [ 10%] (Warmup)

Chain 2: Iteration: 400 / 2000 [ 20%] (Warmup)

Chain 2: Iteration: 600 / 2000 [ 30%] (Warmup)

Chain 2: Iteration: 800 / 2000 [ 40%] (Warmup)

Chain 2: Iteration: 1000 / 2000 [ 50%] (Warmup)

Chain 2: Iteration: 1001 / 2000 [ 50%] (Sampling)

Chain 2: Iteration: 1200 / 2000 [ 60%] (Sampling)

Chain 2: Iteration: 1400 / 2000 [ 70%] (Sampling)

Chain 2: Iteration: 1600 / 2000 [ 80%] (Sampling)

Chain 2: Iteration: 1800 / 2000 [ 90%] (Sampling)

Chain 2: Iteration: 2000 / 2000 [100%] (Sampling)

Chain 2:

Chain 2: Elapsed Time: 49.423 seconds (Warm-up)

Chain 2: 21.946 seconds (Sampling)

Chain 2: 71.369 seconds (Total)

Chain 2:

SAMPLING FOR MODEL '93a90408567ae7343eea598de7d7e540' NOW (CHAIN 3).

Chain 3:

Chain 3: Gradient evaluation took 0 seconds

Chain 3: 1000 transitions using 10 leapfrog steps per transition would take 0 seconds.

Chain 3: Adjust your expectations accordingly!

Chain 3:

Chain 3:

Chain 3: Iteration: 1 / 2000 [ 0%] (Warmup)

Chain 3: Iteration: 200 / 2000 [ 10%] (Warmup)

Chain 3: Iteration: 400 / 2000 [ 20%] (Warmup)

Chain 3: Iteration: 600 / 2000 [ 30%] (Warmup)

Chain 3: Iteration: 800 / 2000 [ 40%] (Warmup)

Chain 3: Iteration: 1000 / 2000 [ 50%] (Warmup)

Chain 3: Iteration: 1001 / 2000 [ 50%] (Sampling)

Chain 3: Iteration: 1200 / 2000 [ 60%] (Sampling)

Chain 3: Iteration: 1400 / 2000 [ 70%] (Sampling)

Chain 3: Iteration: 1600 / 2000 [ 80%] (Sampling)

Chain 3: Iteration: 1800 / 2000 [ 90%] (Sampling)

Chain 3: Iteration: 2000 / 2000 [100%] (Sampling)

Chain 3:

Chain 3: Elapsed Time: 44.674 seconds (Warm-up)

Chain 3: 18.936 seconds (Sampling)

Chain 3: 63.61 seconds (Total)

Chain 3:

SAMPLING FOR MODEL '93a90408567ae7343eea598de7d7e540' NOW (CHAIN 4).

Chain 4:

Chain 4: Gradient evaluation took 0.001 seconds

Chain 4: 1000 transitions using 10 leapfrog steps per transition would take 10 seconds.

Chain 4: Adjust your expectations accordingly!

Chain 4:

Chain 4:

Chain 4: Iteration: 1 / 2000 [ 0%] (Warmup)

Chain 4: Iteration: 200 / 2000 [ 10%] (Warmup)

Chain 4: Iteration: 400 / 2000 [ 20%] (Warmup)

Chain 4: Iteration: 600 / 2000 [ 30%] (Warmup)

Chain 4: Iteration: 800 / 2000 [ 40%] (Warmup)

Chain 4: Iteration: 1000 / 2000 [ 50%] (Warmup)

Chain 4: Iteration: 1001 / 2000 [ 50%] (Sampling)

Chain 4: Iteration: 1200 / 2000 [ 60%] (Sampling)

Chain 4: Iteration: 1400 / 2000 [ 70%] (Sampling)

Chain 4: Iteration: 1600 / 2000 [ 80%] (Sampling)

Chain 4: Iteration: 1800 / 2000 [ 90%] (Sampling)

Chain 4: Iteration: 2000 / 2000 [100%] (Sampling)

Chain 4:

Chain 4: Elapsed Time: 54.744 seconds (Warm-up)

Chain 4: 20.939 seconds (Sampling)

Chain 4: 75.683 seconds (Total)

Chain 4:

Family: gaussian

Links: mu = identity; sigma = log

Formula: y ~ 1 + x + (1 | s | school)

sigma ~ 1 + x + (1 | s | school)

Data: mydata (Number of observations: 2500)

Draws: 4 chains, each with iter = 2000; warmup = 1000; thin = 1;

total post-warmup draws = 4000

Group-Level Effects:

~school (Number of levels: 100)

Estimate Est.Error l-95% CI u-95% CI Rhat Bulk_ESS Tail_ESS

sd(Intercept) 0.19 0.02 0.15 0.24 1.00 1552 2474

sd(sigma_Intercept) 0.11 0.02 0.06 0.16 1.01 1333 1396

cor(Intercept,sigma_Intercept) 0.32 0.18 -0.04 0.67 1.00 2180 2381

Population-Level Effects:

Estimate Est.Error l-95% CI u-95% CI Rhat Bulk_ESS Tail_ESS

Intercept -0.03 0.02 -0.07 0.02 1.00 2188 2671

sigma_Intercept -0.39 0.02 -0.43 -0.36 1.00 3600 3190

x 0.70 0.01 0.67 0.73 1.00 6686 3234

sigma_x 0.03 0.02 -0.00 0.05 1.00 5863 3170

Draws were sampled using sampling(NUTS). For each parameter, Bulk_ESS

and Tail_ESS are effective sample size measures, and Rhat is the potential

scale reduction factor on split chains (at convergence, Rhat = 1).

The model ran four chains each with 1000 warmup (burn-in) iterations and 1000 monitoring iterations. Recall that the model is a reparametrized version of model S1. The outputs additionally shows that the elements of the random effect covariance matrix used in this alternative parameterizations are presented as SDs and correlations rather than as variances and covariances. We can recover the random effect variances of this alternative parameterization by squaring the random effect SDs. The random effect covariance of this alternative parameterization can be recovered by multiplying the random effect correlation by the two random effect SDs. We can then recover the parameter and random effect values associated with the parameterization S1 using the transformations listed previously. All these calculations are best applied to the underlying chains rather than the means which are displayed in the output. Having carried out these steps, the results are as follows.

|  | True value | Est. | SE |
| --- | --- | --- | --- |
| $\beta_{0}$ | 0.000 | -0.028 | 0.024 |
| $\beta_{1}$ | 0.700 | 0.701 | 0.014 |
| $\sigma_{u}^{2}$ | 0.050 | 0.038 | 0.008 |
| $\alpha_{0}$ | -0.800 | -0.784 | 0.037 |
| $\alpha_{1}$ | 0.050 | 0.050 | 0.030 |
| $\sigma_{v}^{2}$ | 0.050 | 0.054 | 0.022 |
| $\sigma_{uv}$ | 0.025 | 0.014 | 0.009 |

The parameter estimates are similar to their true values and to those provided by bayesmh in Stata (above) and MixWILD (below).

## MixWILD

The MixWILD software (Dzubur et al., 2020) is freely available at the software website <https://reach-lab.github.io/MixWildGUI/>. MixWILD fits models using maximum likelihood estimation via adaptive quadrature. We use the default estimation options which specifies 11 quadrature points. We encourage readers to consult the comprehensive documentation for further details.

The MixWILD software specifies model S1 using the following alternative parameterization.

$y_{ij}=\beta_{0}+\beta_{1}x_{ij}+u_{j}+e_{ij}$ (S3)

$$\ln\left( \sigma_{u}^{2} \right)=\gamma_{0}$$

$$\ln\left( \sigma_{e,ij}^{2} \right)=\alpha_{0}+\alpha_{1}x_{ij}+\tau\frac{u_{j}}{\sigma_{u}}+v_{j}^{'}$$

$$\left( \begin{matrix} u_{j} \\ v_{j}^{'} \end{matrix} \right) \sim N\left\{ \left( \begin{matrix} 0 \\ 0 \end{matrix} \right),\left( \begin{matrix} \sigma_{u}^{2} & \\ 0 & \sigma_{v^{'}}^{2} \end{matrix} \right) \right\}$$

The mean function random effect variance is specified on the log scale $\gamma_{0}$. The mean and residual function random effects are assumed independent. The association between the mean and residual variance functions is instead allowed for via entering the mean function random effect as a standardized latent covariate in the residual variance function and estimating its regression coefficient $\tau$. Fortunately, the parameter and random effect values of the original parameterization are easily recovered as follows $\sigma_{u}^{2}=\exp\left( \gamma_{0} \right)$, $v_{j}=\frac{u_{j}}{\sigma_{u}}+v_{j}^{'}$, $\sigma_{v}^{2}=\tau^{2}+\sigma_{v^{'}}^{2}$, $\sigma_{uv}=\tau\sigma_{u}$.

The point-and-click instructions to specify and fit this model are as follows.

- Open **MixWILD** by double clicking on the MixWILD icon

MixWILD will open and you will see the following **Model Configuration** tab


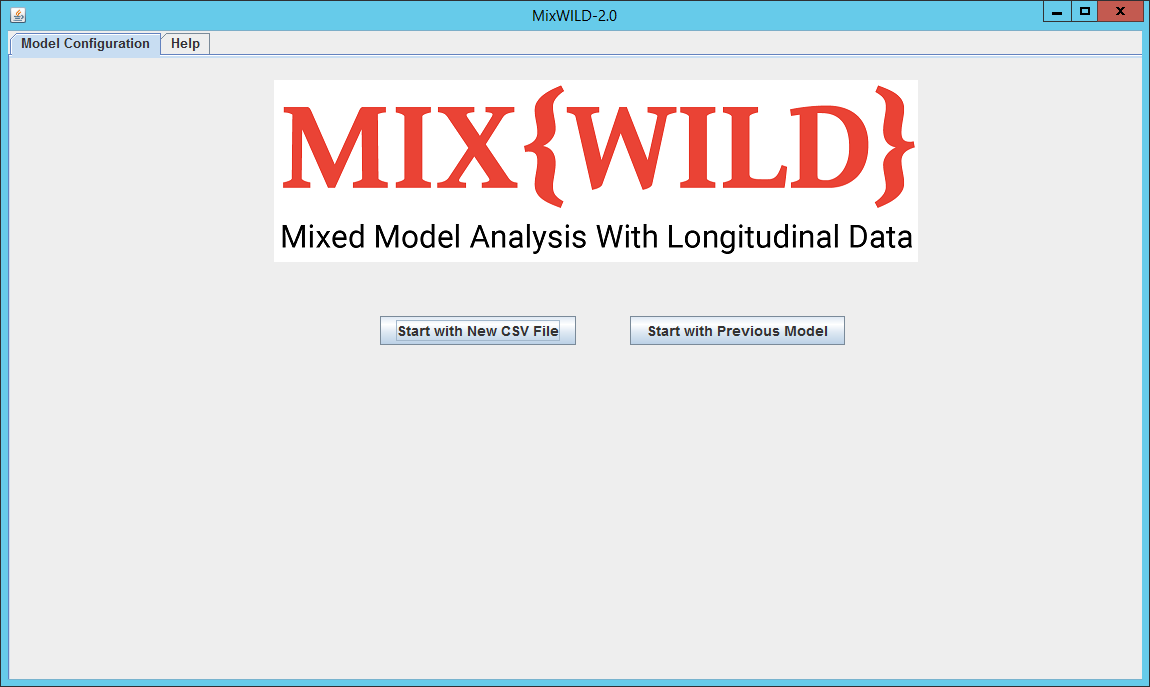


- Click **Start with New CSV File**

An **Open** file window will appear

- Navigate to wherever you have saved **data.csv** and click **Open** (please make sure there are no spaces in the file address!)

The **Model Configuration** tab will update to match that shown below


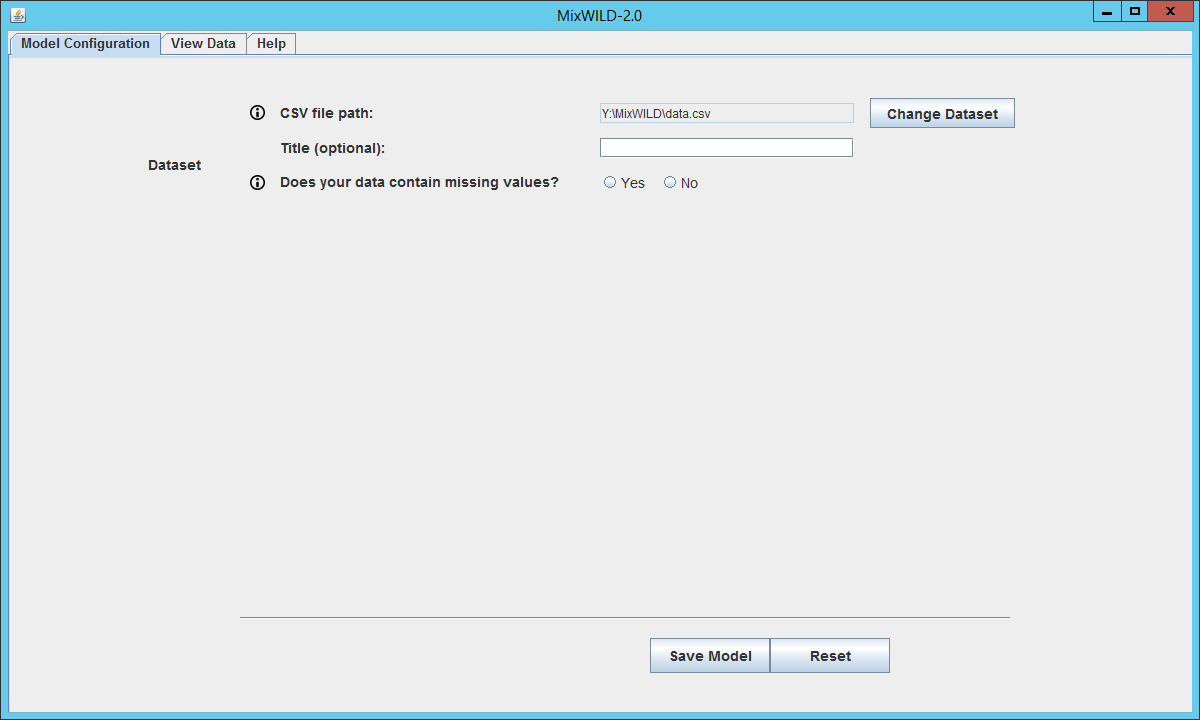


- Next to **Does your data contain missing values?**, select **No**

The **Model Configuration** tab will update to reveal more options as shown below.


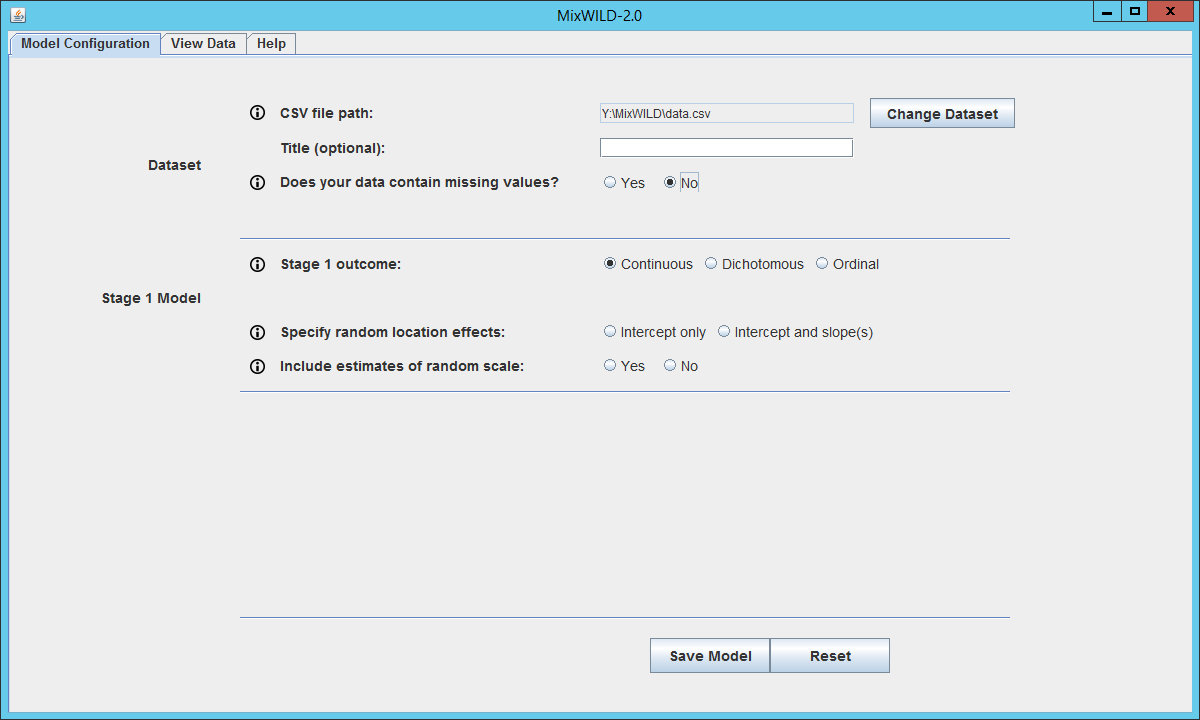


- Next to **Specify random location effects**, select **Intercept only**
- Next to **Include estimates of random scale**, select **Yes**

The **Model Configuration** tab will update to reveal more options as shown below.


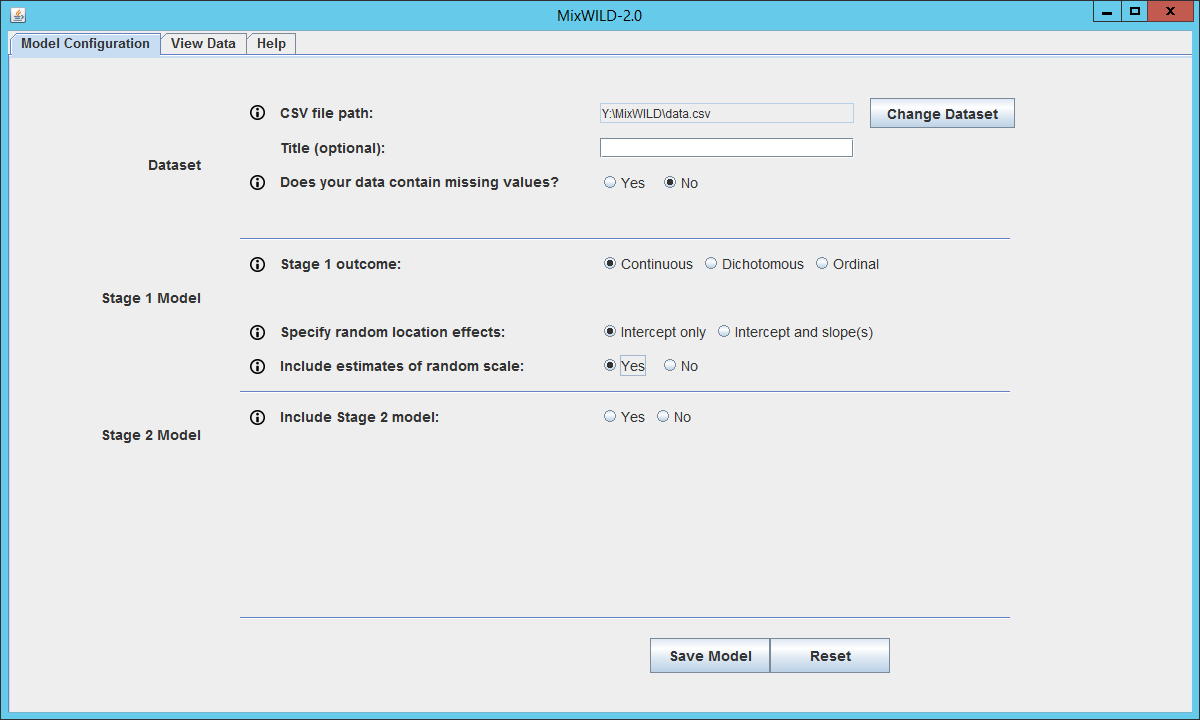


- Next to **Include Stage 2 model**, select **No**
- Check that the **Model Configuration** tab matches that shown below, then click **Continue**


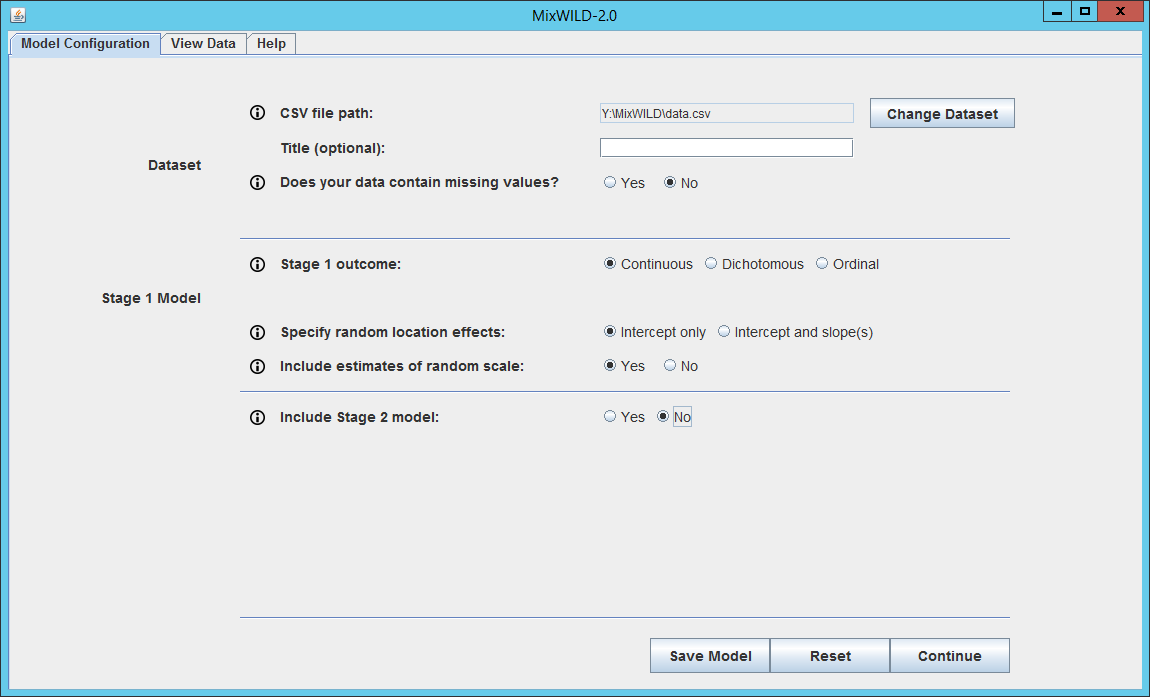


The **Stage 1 Configuration** tab will then be displayed


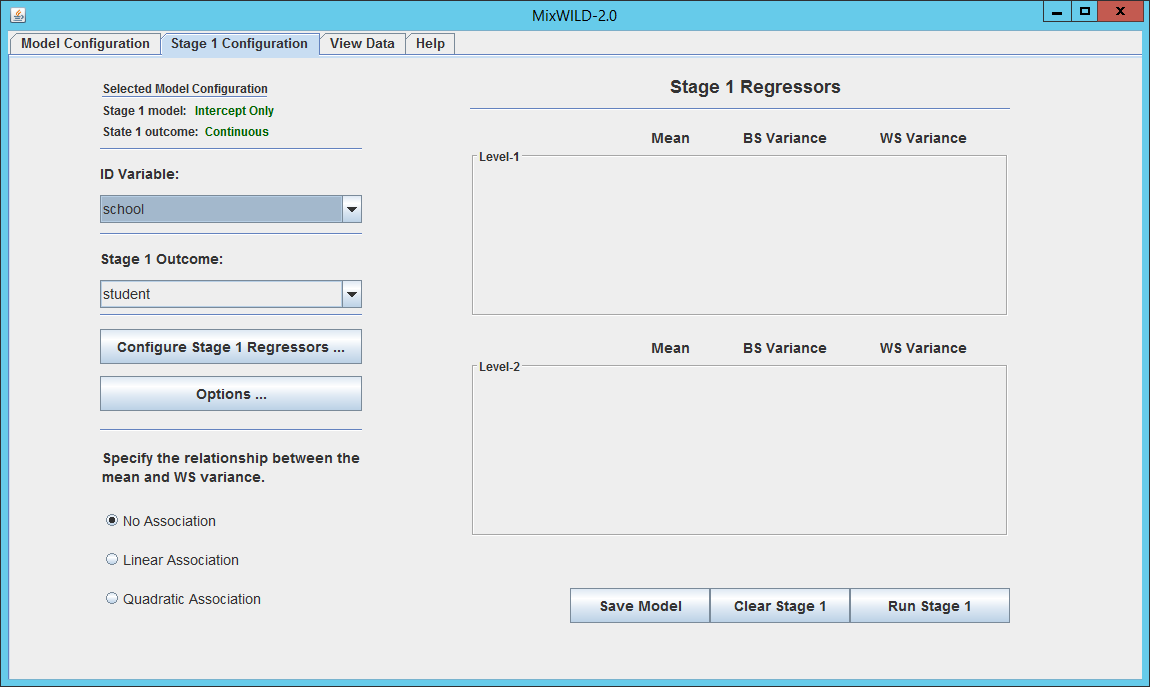


- Under the **Stage 1 Outcome** drop-down list, select **y**
- Click **Configure State 1 Regressors …**

The **Add Stage 1 Regressors** window will open


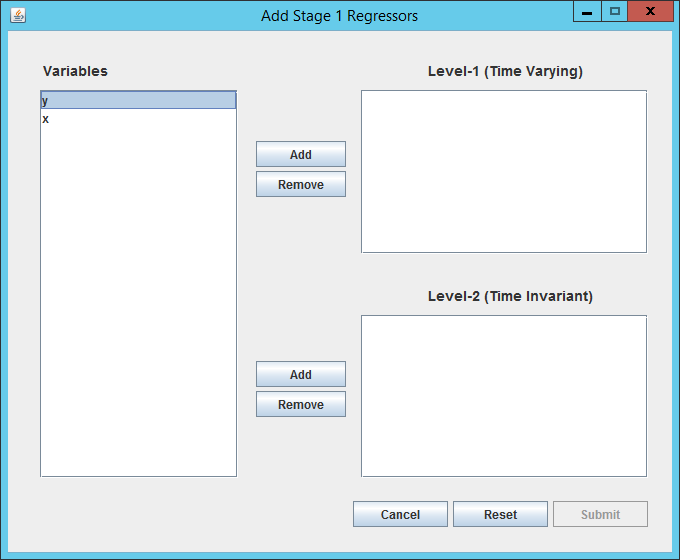


- In the **Variables** box, select **x**
- Next to the **Level-1 (Time Varying)** box, click **Add**
- Check that the **Add Stage 1 Regressors** tab matches that shown below, then Click **Submit**


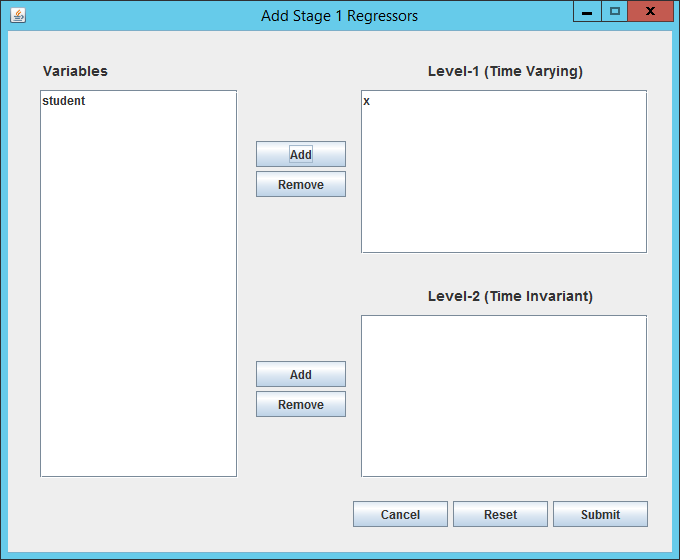


You will once again see the **Stage 1 Configuration** tab


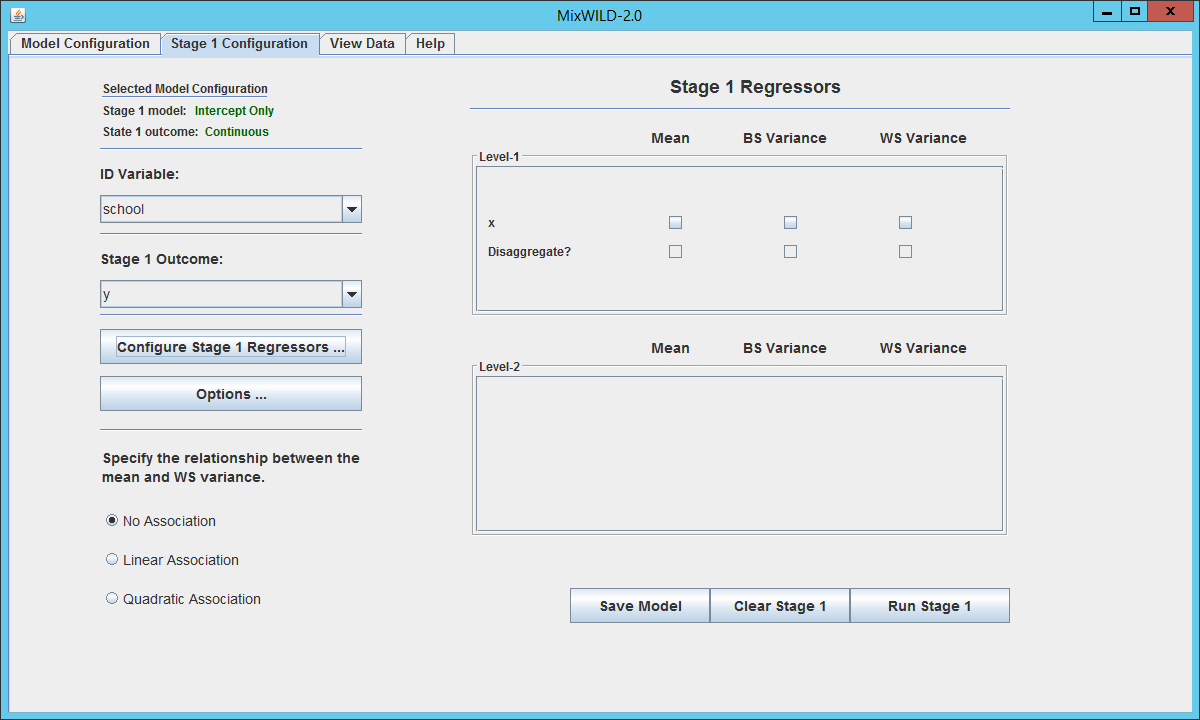


- Under **Stage 1 Regressors**, under **Level-1**, check the two checkboxes with row label **x** and column labels **Mean** and **WS Variance**
- Under **Specify the relationship between the mean and WS variance**, select **Linear Association**
- Check the **Stage 1 Configuration** tab matches that shown below, then click **Run Stage 1**


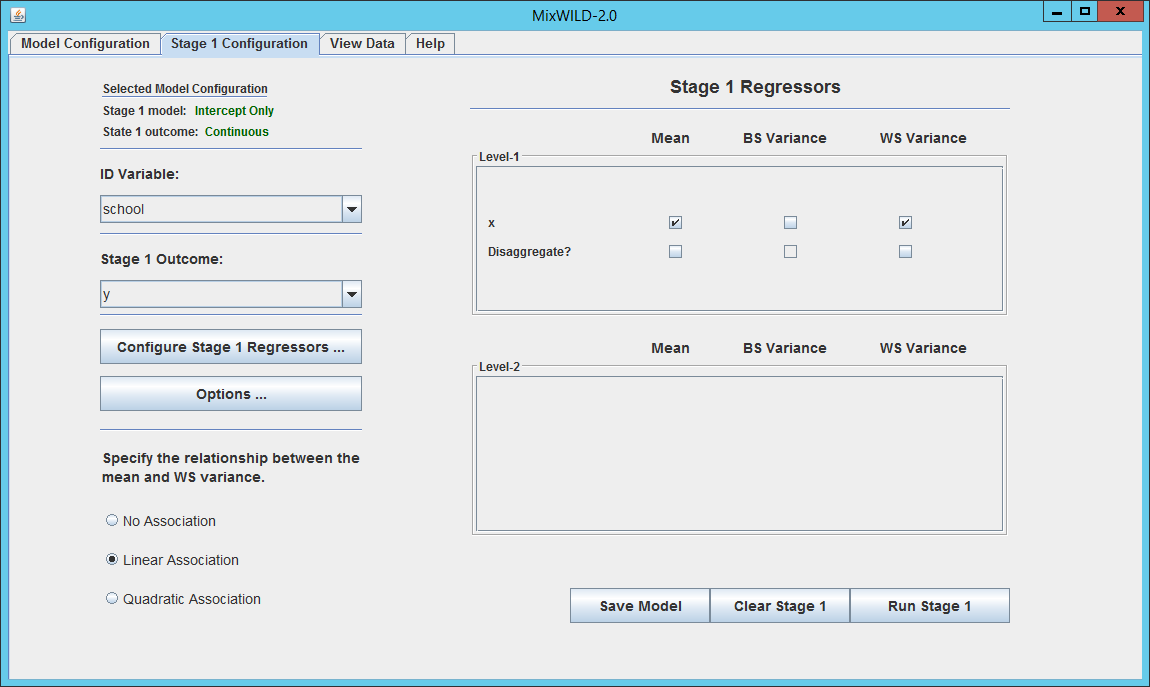


The following **Definition File Preview** window will open


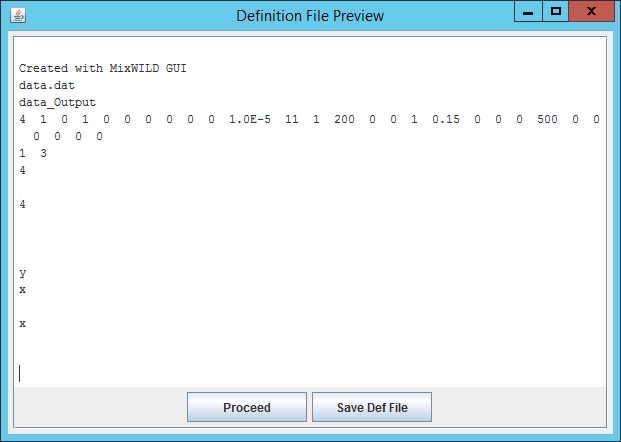


- Click **Proceed**

The following **Please wait … window** will appear


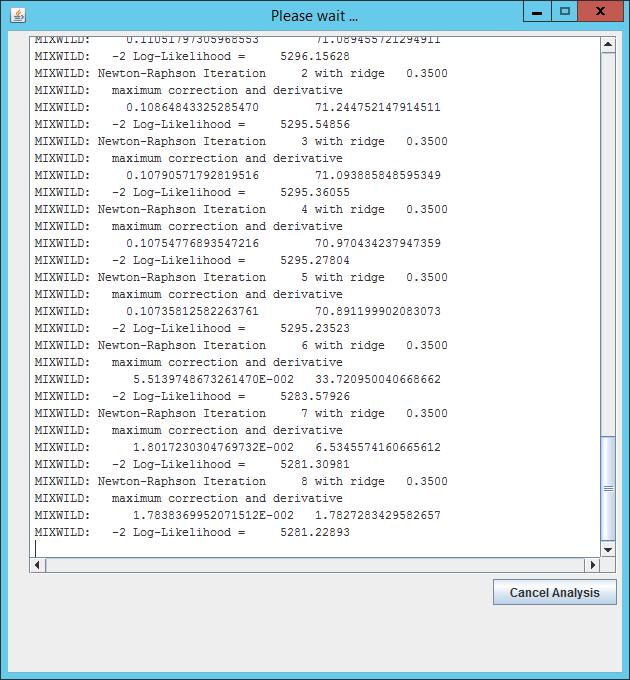


Once the estimation has completed, the window will automatically close and you will see the following **Stage 1 Results** tab


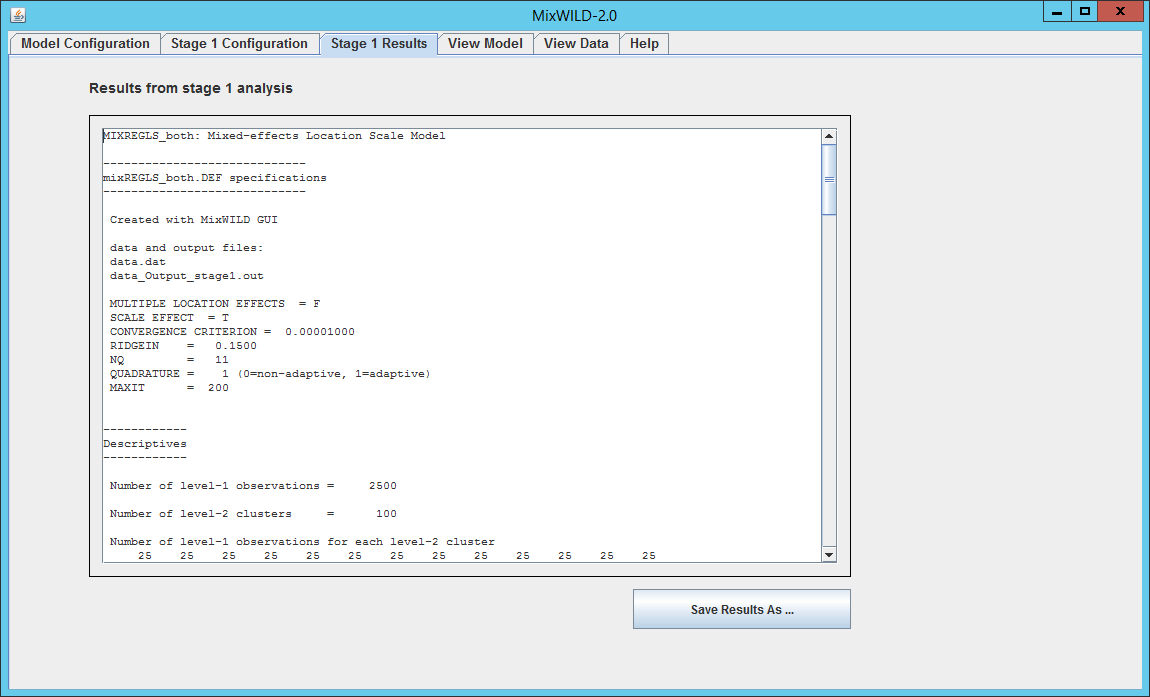


The results presented in this window are pasted and discussed below

MIXREGLS_both: Mixed-effects Location Scale Model

-----------------------------

mixREGLS_both.DEF specifications

-----------------------------

Created with MixWILD GUI

data and output files:

data.dat

data_Output_stage1.out

MULTIPLE LOCATION EFFECTS = F

SCALE EFFECT = T

CONVERGENCE CRITERION = 0.00001000

RIDGEIN = 0.1500

NQ = 11

QUADRATURE = 1 (0=non-adaptive, 1=adaptive)

MAXIT = 200

------------

Descriptives

------------

Number of level-1 observations = 2500

Number of level-2 clusters = 100

Number of level-1 observations for each level-2 cluster

25 25 25 25 25 25 25 25 25 25 25 25 25

25 25 25 25 25 25 25 25 25 25 25 25 25

25 25 25 25 25 25 25 25 25 25 25 25 25

25 25 25 25 25 25 25 25 25 25 25 25 25

25 25 25 25 25 25 25 25 25 25 25 25 25

25 25 25 25 25 25 25 25 25 25 25 25 25

25 25 25 25 25 25 25 25 25 25 25 25 25

25 25 25 25 25 25 25 25 25

Dependent variable

mean min max std dev

----------------------------------------------------------------

y -0.0366 -3.4413 3.6288 1.0033

Mean model covariates

mean min max std dev

----------------------------------------------------------------

intercept 1.0000 1.0000 1.0000 0.0000

x -0.0124 -4.5322 3.4890 1.0074

BS variance model covariates

mean min max std dev

----------------------------------------------------------------

intercept 1.0000 1.0000 1.0000 0.0000

WS variance model covariates

mean min max std dev

----------------------------------------------------------------

intercept 1.0000 1.0000 1.0000 0.0000

x -0.0124 -4.5322 3.4890 1.0074

------------------------------

Model without Scale Parameters

------------------------------

Total Iterations = 4

Final Ridge value = 0.2

Log Likelihood = -2649.111

Akaike's Information Criterion = -2653.111

Schwarz's Bayesian Criterion = -2658.321

==> multiplied by -2

Log Likelihood = 5298.221

Akaike's Information Criterion = 5306.221

Schwarz's Bayesian Criterion = 5316.642

Variable Estimate AsymStdError z-value p-value

---------------- ------------ ------------ ------------ ------------

BETA (regression coefficients)

intercept -0.02799 0.02358 -1.18736 0.23509

x 0.70113 0.01453 48.24144 0.00000

ALPHA (BS variance parameters: log-linear model)

intercept -3.29934 0.21377 -15.43434 0.00000

TAU (WS variance parameters: log-linear model)

intercept -0.76221 0.02887 -26.40572 0.00000

---------------------------

Model WITH Scale Parameters

---------------------------

Total Iterations = 13

Final Ridge value = 0.0

Log Likelihood = -2646.541

Akaike's Information Criterion = -2651.541

Schwarz's Bayesian Criterion = -2658.054

==> multiplied by -2

Log Likelihood = 5293.081

Akaike's Information Criterion = 5303.081

Schwarz's Bayesian Criterion = 5316.107

Variable Estimate AsymStdError z-value p-value

---------------- ------------ ------------ ------------ ------------

BETA (regression coefficients)

intercept -0.02778 0.02344 -1.18525 0.23592

x 0.70271 0.01448 48.51461 0.00000

ALPHA (BS variance parameters: log-linear model)

intercept -3.31776 0.21505 -15.42792 0.00000

TAU (WS variance parameters: log-linear model)

intercept -0.76310 0.02887 -26.42848 0.00000

x 0.06373 0.02812 2.26655 0.02342

-----------------------

Model WITH RANDOM Scale

-----------------------

Total Iterations = 13

Final Ridge value = 0.0

Log Likelihood = -2639.033

Akaike's Information Criterion = -2646.033

Schwarz's Bayesian Criterion = -2655.151

==> multiplied by -2

Log Likelihood = 5278.066

Akaike's Information Criterion = 5292.066

Schwarz's Bayesian Criterion = 5310.302

Variable Estimate AsymStdError z-value p-value

---------------- ------------ ------------ ------------ ------------

BETA (regression coefficients)

intercept -0.02783 0.02328 -1.19549 0.23190

x 0.70159 0.01432 48.98862 0.00000

ALPHA (BS variance parameters: log-linear model)

intercept -3.33908 0.21800 -15.31721 0.00000

TAU (WS variance parameters: log-linear model)

intercept -0.78759 0.03724 -21.15057 0.00000

x 0.05011 0.02971 1.68650 0.09170

Random scale standard deviation

Std Dev 0.21398 0.04480 4.77662 0.00000

Random location (mean) effect on WS variance

Loc Eff 0.07740 0.04367 1.77238 0.07633

BS variance ratios and 95% CIs

------------------------------

Variable Ratio Lower Upper

---------------- ------------ ------------ ------------

ALPHA (BS variance parameters: log-linear model)

intercept 0.03547 0.02314 0.05438

WS variance ratios and 95% CIs

------------------------------

Variable Ratio Lower Upper

---------------- ------------ ------------ ------------

TAU (WS variance parameters: log-linear model)

intercept 0.45494 0.42292 0.48938

x 1.05138 0.99191 1.11443

Random location (mean) effect on WS variance

Location Effect 1.08047 0.99184 1.17702

Random scale standard deviation

Std Dev 1.23859 1.13448 1.35226

The MixWILD output is presented in five sections. The first four sections present the estimation options, descriptive statistics, and the results of two simpler versions of the full model used to generating starting values for the full model. The fifth and final block of output titled “Model WITH RANDOM Scale” presents the results for the full model. Recall that the model is a reparametrized version of model S1. The output additionally shows that the residual variance function random effect variance used in this alternative parameterization is presented as a SD rather than a variance. We can recover the random effect variance of this alternative parameterization by squaring the random effect SD. We can then recover the parameter and random effect values associated with the original S1 parameterization using the transformations listed previously. The corresponding standard errors can be recovered via the delta method. Having carried out these steps, the results presented in tabular form are as follows

|  | True value | Est. | SE |
| --- | --- | --- | --- |
| $\beta_{0}$ | 0.000 | -0.028 | 0.023 |
| $\beta_{1}$ | 0.700 | 0.701 | 0.014 |
| $\sigma_{u}^{2}$ | 0.050 | 0.035 | 0.008 |
| $\alpha_{0}$ | -0.800 | -0.785 | 0.037 |
| $\alpha_{1}$ | 0.050 | 0.050 | 0.030 |
| $\sigma_{v}^{2}$ | 0.050 | 0.052 | 0.020 |
| $\sigma_{uv}$ | 0.025 | 0.015 | 0.008 |

The parameter estimates are similar to their true values and to those provide by bayesmh in Stata and brms in R (above).

# References

Bürkner, P.-C. (2017). brms: An R Package for Bayesian Multilevel Models Using Stan. *Journal of Statistical Software*, *80(1)*, 1–28.

Bürkner P.-C. (2018). Advanced Bayesian Multilevel Modeling with the R Package brms. *The R Journal*, *10(1)*, 395–411.

Dzubur, E., Ponnada, A., Nordgren, R., Yang, C. H., Intille, S., Dunton, G., & Hedeker, D. (2020). MixWILD: A program for examining the effects of variance and slope of time-varying variables in intensive longitudinal data. *Behavior Research Methods*, 1-25.

Stan Development Team (2021). *Stan Modeling Language User’s Guide and Reference Manual, Version 2.27.0*. URL: <http://mc-stan.org>.

StataCorp. (2021). *Stata 17 Bayesian Analysis Reference Manual*. College Station, TX: Stata Press.
